# An epidemic-economic model for COVID-19

**DOI:** 10.3934/mbe.2022449

**Published:** 2022-07-04

**Authors:** Jie Bai, Xiunan Wang, Jin Wang

**Affiliations:** 1 School of Mathematics and Statistics, Liaoning University, Shenyang 110036, China; 2 Department of Mathematics, University of Tennessee at Chattanooga, Chattanooga, TN 37403, USA

**Keywords:** COVID-19, compartmental modeling, data fitting, equilibrium analysis, the epidemic and economic consequences

## Abstract

In this paper, we propose a new mathematical model to study the epidemic and economic consequences of COVID-19, with a focus on the interaction between the disease transmission, the pandemic management, and the economic growth. We consider both the symptomatic and asymptomatic infections and incorporate the effectiveness of disease control into the respective transmission rates. Meanwhile, the progression of the pandemic and the evolution of the susceptible, infectious and recovered population groups directly impact the mitigation and economic development levels. We fit this model to the reported COVID-19 cases and unemployment rates in the US state of Tennessee, as a demonstration of a real-world application of the modeling framework.

## 1. Introduction

The coronavirus disease 2019 (COVID-19) led to high morbidity and mortality rates throughout the world and created unprecedented challenges in public health and the global economy. Two years have passed since the World Health Organization (WHO) officially declared COVID-19 as a global pandemic in March 2020, but many nations are still struggling with the spread of the infection. The emergence of several new SARS-CoV-2 variants adds further uncertainty to the progression of the pandemic. As of March 2022, more than 470 million cases and 6 million deaths were reported in 225 countries and territories [1].

During the first few months of the pandemic, the rapidity of how the SARS-CoV-2 spread across the world caught most nations unawares and poorly prepared to contain the disease outbreaks and treat the most severe cases [2,3]. The situation was compounded by the lack of widespread testing, absence of COVID-19 vaccines, and unavailability of effective treatment modalities [2]. Furthermore, since the prevalence of COVID-19 among those with mild or no symptoms can be considerably high, tracking the transmission of the disease had proved difficult [4]. Nevertheless, fundamental public health mitigation strategies were implemented which included, but were not limited to, personal and environmental hygienic practices, isolation and quarantine of known cases, contact tracing, social distancing, closure of non-essential businesses and services, and shelter-in-place orders [5, 6]. Such mitigation strategies appeared effective in slowing the transmission of the virus and reducing the number of cases in communities [7, 8].

The spread of COVID-19 also caused severe downturn and huge uncertainty for the economy throughout the world [9–13]. In particular, during the “Stay-at-Home” period (from March to May in 2020) in the US, non-essential businesses were shut down and the US experienced a sharp increase in unemployment as of the end of March 2020, representing a 9.5% unemployment rate [14]. This increase, up from 3.5% in February 2020, was just one indication on the breadth of the economic upheaval caused by COVID-19. Meanwhile, the US Department of Commerce reported that total retail sales in March 2020 plunged by 8.7 percent from the previous month, the largest decline in the nearly three decades the government had tracked the data [15]. The impact on the global economic and financial system were estimated as being at least a 3% reduction in gross domestic product (GDP), with global financial markets experiencing dramatic instability as seen in the stock markets, assets, and risk markets [16].

A large body of experimental, clinical and theoretical studies have been generated to better understand COVID-19 and its consequences, and explore more effective intervention strategies (see reviews [12,17–20] and references therein). Particularly, many mathematical and statistical models have been published to study the transmission and spread of COVID-19 [21–33], as well as the effects of COVID-19 vaccination [34–38]. Meanwhile, a number of computational and quantitative studies have considered the impact of COVID-19 on the economic development. For example, de la Fuente-Mella et al. statistically evaluated the effects of the COVID-19 pandemic on the economy of several countries [39]. Chen et al. proposed a network-based epidemic-economic model to estimate the direct impact of labor supply shock to each sector arising from morbidity, mortality, and lockdown [40]. Altig et al. analyzed several economic uncertainty indicators for the US and UK before and during the COVID-19 pandemic [41]. Eichenbaum et al. combined a canonical epidemiology model with macroeconomic models to study the interaction between economic decisions and epidemics [9]. Jena et al. developed a multilayer artificial neural network model to forecast the impact of COVID-19 on the GDP of eight countries including the US [42]. Xiang et al. combined economic theory with an epidemiological model to explore the long-term impact of the pandemic on economic growth and the effects of different policy responses [43]. Dimarco et al. proposed a wealth transfer model to compute the Gini-index in the presence of a pandemic, with an observation of the emergence of economic inequalities [44]. In addition, Auld and Toxvaerd estimated behavioral responses to the global rollout of COVID-19 vaccines and found that countries with earlier and higher vaccination coverage strongly tended to be richer [45].

In spite of these studies, a fundamental question remains to be answered: How do the COVID-19 transmission and spread, the disease prevention and intervention, and the economic development impact each other within an interconnected triad? The persistence of the pandemic and the wide-ranging slowdown of the global economy at present, long after the onset of the pandemic, underscore the gap between the complex epidemic-economic mechanisms associated with COVID-19 and our current understanding of these phenomena.

In this paper, we seek to partially address this fundamental question by formulating a new mathematical model to quantify the economic impact of COVID-19 and investigate the interplay between the epidemic progression, the economic growth, and the disease management. We consider both the symptomatic and asymptomatic infections in this model, and incorporate the effectiveness of disease control into the respective transmission rates. Meanwhile, factors associated with economics and epidemiology are both included into the model, where the level of pandemic mitigation is negatively correlated to that of economic development. Additionally, the progression of the pandemic and the evolution of the susceptible, infectious and recovered population groups directly impact the mitigation and economic development levels.

To demonstrate a real-world application of this modeling framework, we study the epidemic and economic situations in the US state of Tennessee in 2020. We utilize the unemployment rate, defined by the number of people who are unemployed as a percentage of the labor force, as the key economic indicator in this work. It is known that a high unemployment rate increases economic inequality, generates redistributive pressures, drives people to poverty, and negatively affects long-run economic growth. Our aim is to study how the unemployment rate would intertwine with the mitigation efforts under the impact of COVID-19. We fit our model to the COVID-19 cases and the unemployment rates reported in Tennessee [46, 47], and conduct detailed numerical simulation to examine the relationship between the epidemic spread, the disease management, and the economic development.

The remainder of this manuscript is organized as follows. We present the model formulation in Section 2, with detailed mathematical analysis provided in Appendices A and B. We then conduct data fitting and numerical simulation using the epidemic and economic data from the US state of Tennessee in Section 3. We conclude the paper with some discussion in Section 4.

## 2. Model formulation

We divide the human population into four compartments: the susceptible (*S*), the exposed (*E*), the symptomatic infectious (*I*), and the recovered (*R*). A distinctive feature of COVID-19 is that asymptomatic and pre-symptomatic infection is common [18, 48], so that infected individuals could be contagious even during the incubation period. We thus assume that the exposed individuals are capable of transmitting the disease; basically, they are regarded as pre-symptomatic infectious individuals in this study. To incorporate the impact of mitigation policies on economic development, we introduce two additional variables: the mitigation level/effectiveness, denoted by *M*, and the economic development level, denoted by *C*. We normalize the range of *M* such that 0 ≤ *M* ≤ 1, with *M* = 1 representing the situation with the maximum disease control and *M* = 0 the situation with no disease control at all. Similarly, we normalize *C* to the range between 0 and 1 such that *C* = 1 indicates the maximum level of economic development and *C* = 0 indicates the worst scenario of economic development. We assume that the transmission rates are modulated by the disease control, that the mitigation level is stimulated by the disease prevalence, and that the economic development level depends on the available labor supply. We additionally assume that the disease mitigation level and economic development level are negatively correlated to each other.

The following differential equations represent our epidemic-economic model for COVID-19 transmission dynamics, with descriptions for all the parameters provided in Table 1.


(2.1) $$\frac{dS}{dt}=\lambda -\frac{\beta_{E}}{1+bM}SE-\frac{\beta_{I}}{1+bM}SI-\mu S,\;\frac{dE}{dt}=\frac{\beta_{E}}{1+bM}SE+\frac{\beta_{I}}{1+bM}SI-(\alpha +\mu )E,\;\frac{dI}{dt}=\alpha E-(w+\gamma +\mu )I,\;\frac{dR}{dt}=\gamma I-\mu R,\;\frac{dM}{dt}=\delta +mI-pM-f_{0}C,\;\frac{dC}{dt}=c_{S}S+c_{E}E+c_{R}R-dC-g_{0}M.$$

**Figure 1.**
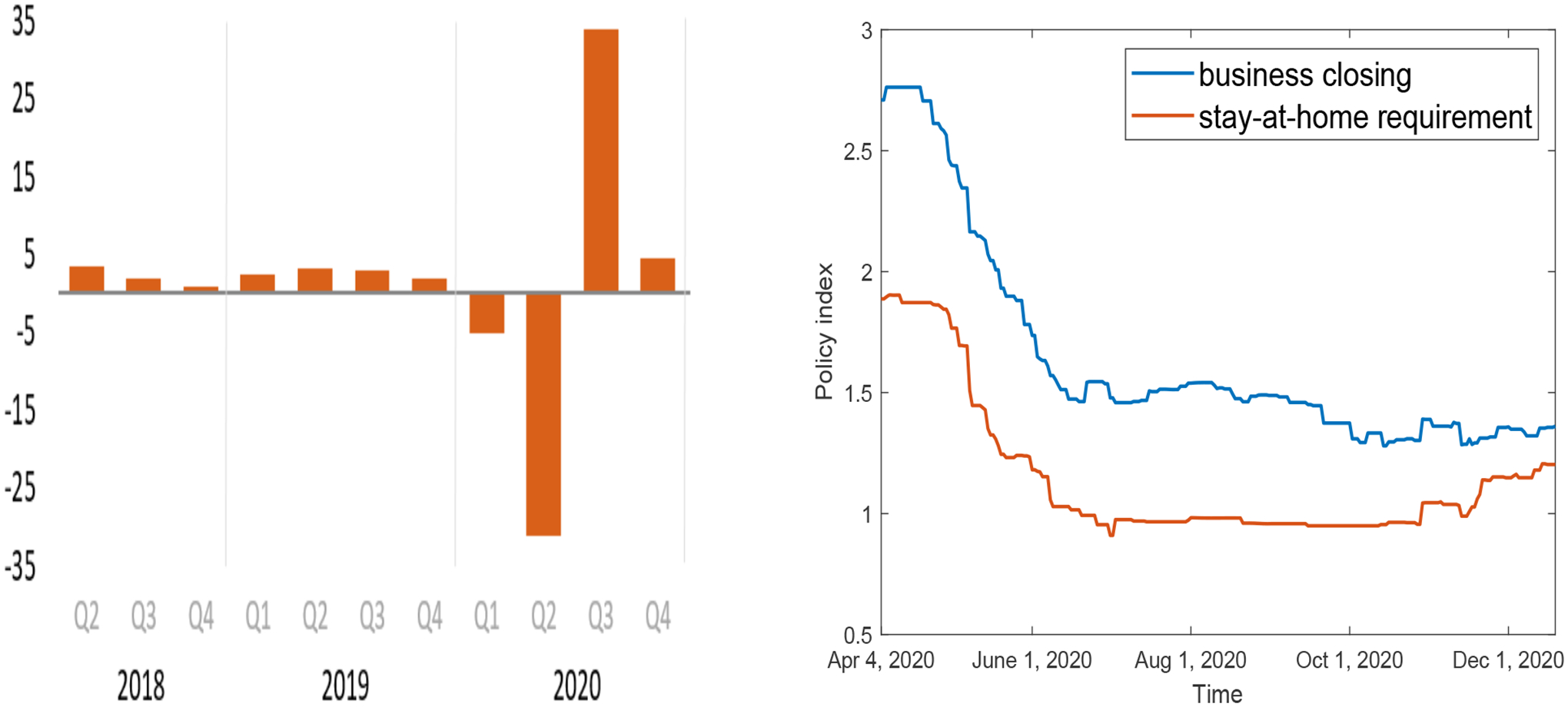
Data suggest a negative correlation between the economic growth and pandemic management in the US. Left: Percent change of the GDP from preceding quarter (source: U.S. Bureau of Economic Analysis [49]); Right: Strength of business closure and stay-at-home requirement (source: Our World in Data [50]).

The last two equations in system (2.1) reflect the negative correlation between the mitigation level and the economic development level; i.e., the increase of one variable would tend to decrease the other. This assumption has been motivated by empirical observations of the pandemic management and economic growth in the US. Figure 1 (left) shows the quarterly percentage change of the GDP in the US from 2018 to 2020 [49]. In particular, under the impact of COVID-19, the GDP in the first quarter of 2020 experienced a reduction of about 5% from the preceding quarter, and the second quarter GDP plunged about 32%. Such reductions were largely due to the closure of businesses, implementation of stay-at-home orders, and other pandemic mitigation strategies that started in March 2020 and extended to May/June 2020. Afterwards, the third quarter saw a large increase of GDP (about 33%) from the previous quarter, in parallel with the re-opening of businesses, removal of the stay-at-home requirement, and other relaxed control measures. The fourth quarter in 2020 continued the trend of GDP growth with a moderate 4% increase. Figure 1 (right) displays the quantification of the business closure and stay-at-home requirement as two representative mitigation policies, where a higher number indicates a stronger effort, for the US in 2020 [50]. We can observe that both policies had the highest strengths in April, which then quickly decreased afterwards, reaching a low point in June/July. For the last two quarters in 2020, the two policy curves stayed at approximately the same levels as that in June, though occasional slight increases ( e.g., July–August, November–December) can be noticed. These figures convey the message that a focus on pandemic management would slow down economic development, whereas an emphasis on economic growth would necessitate policy changes which may subsequently increase the risk of disease transmission and weaken the disease control efforts.

The basic reproduction number of the model is

(2.2) $$R_{0}=R_{E0}+R_{I0}=\frac{\beta_{E}S_{0}}{\left(1+bM_{0}\right)(\alpha +\mu )}+\frac{\alpha \beta_{I}S_{0}}{\left(1+bM_{0}\right)(\alpha +\mu )(w+\gamma +\mu )},$$

where *S*_0_ and *M*_0_ are the respective values of *S* and *M* at the disease-free equilibrium. The derivation of $R_{0}$ is provided in Appendix A. We see that $R_{0}$ includes two parts, representing the contributions from two respective transmission routes: one starts from the exposed, or asymptomatic infectious, individuals (denoted as $R_{E0}$), and the other starts from the symptomatic infectious individuals (denoted as $R_{I0}$). We refer to $R_{E0}$ as the asymptomatic reproduction number, and $R_{I0}$ as the symptomatic reproduction number.

We have also conducted an equilibrium analysis of system (2.1) by assuming that all the parameters are constants. The details are provided in Appendix B. In particular, the analysis indicates that in such a homogeneous setting, the disease will persist and become endemic in the long run if $R_{0}>1$. In practical applications, however, public health and economic policies typically change with time, and disease control strategies are adjusted accordingly. These variations, subsequently, impact the progression of the epidemic and lead to more complex situations than a homogeneous scenario. In the next section, we will use numerical simulation to explore such time-dependent behaviors of the system dynamics.

## 3. Simulation results

We apply our model to the COVID-19 epidemic in the US state of Tennessee. We use the unemployment rate as the key economic indicator to infer the economic development level. Specifically, we set *C*(*t*) = 1 − *U*(*t*) in our model, where *U*(*t*) denotes the unemployment rate at time *t*. We have collected daily and weekly data for the COVID-19 cases and the unemployment rates in Tennessee [46, 47], from March 28, 2020 to December 31, 2020. We fit our model to such data and conduct numerical simulation to examine the interaction between the epidemic spread, the disease management, and the economic development.

Since the epidemic progression of COVID-19 exhibits very different behaviors at different times, we divide the entire time interval (from March 28 to December 31, 2020) into five consecutive periods:

- Period 1: From March 28 to April 30
- Period 2: From May 1 to May 21
- Period 3: From May 22 to July 20
- Period 4: From July 21 to September 30
- Period 5: From October 1 to December 31

For each period, we conduct separate data fitting, with model parameters and initial conditions defined in Table 1. A gradient-based nonlinear constrained optimization procedure, implemented through the Matlab function *fmincon*, is employed to fit all the model parameters except the population influx rate *λ*, the recovery rate *γ*, the incubation period *α*^−1^, and the natural and disease-induced death rates *μ* and *ω*, whose values are prescribed from the literature. We have found that this approach performs sufficiently well in our study. Nevertheless, we mention that many other data fitting techniques can be possibly applied, including some more sophisticated approaches such as a two-level fitting method presented in [52].

The fitting in different periods results in different estimates of parameter values in the model. All these fitted parameter values are listed in Table 2. We focus our attention on the data fitting and numerical simulation in Period 1, with details provided in Section 3.1. The analysis and discussion can be similarly applied to the other periods. A summary of the fitting and simulation results for Periods 2–5 is provided in Section 3.2.

### 3.1. Fitting and simulation in Period 1

#### 3.1.1. Data fitting

**Figure 2.**
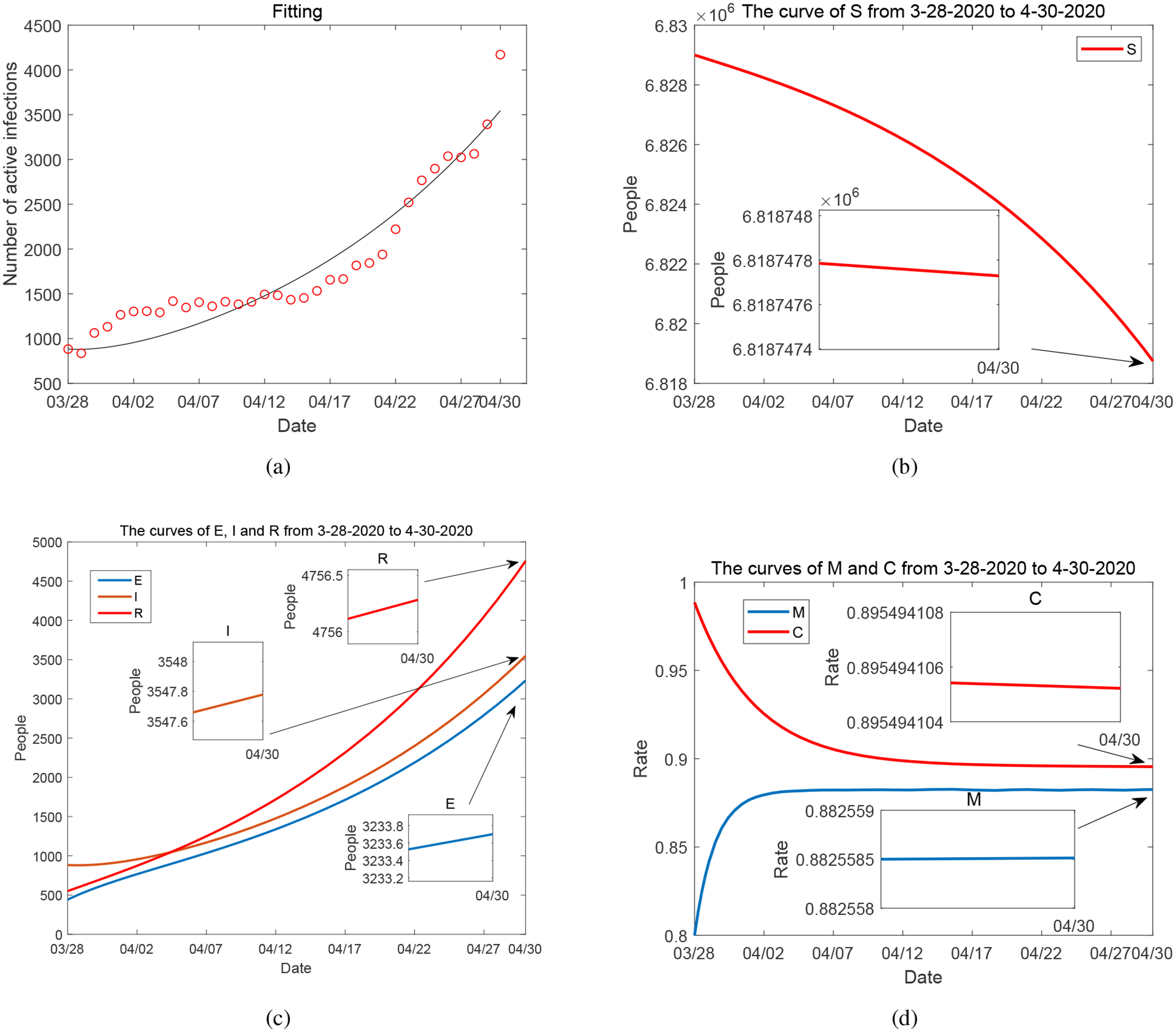
(a) Data fitting for active COVID-19 cases in Tennessee during Period 1 (from March 28, 2020 to April 30, 2020): circles represent the reported data and solid line represents the fitting result. (b)–(d): Curves of *S*, *E*, *I*, *R*, *M* and *C* from numerical simulation.

Period 1, from March 28, 2020 to April 30, 2020, represents an initial phase of the COVID-19 epidemic in Tennessee (as well as in the entire US), with the number of infections fast increasing. Figure 2(a) shows our model fitting to the number of daily active cases reported by the Tennessee Department of Health [46], where we see that the number of active infections increased from below 1000 in the beginning to above 4000 at the end of the period. We observe a reasonably good match between our simulation result and the reported data.

Based on the estimated parameter values from Table 2, we evaluate the basic reproduction number from Eq (2.2):

(3.1) $$R_{10}=R_{1E0}+R_{1I0}=0.23+1.78=2.01,$$

where the subscript ‘1’ indicates the association with Period 1. Clearly, $R_{10}>1$, which accounts for the fast increase of infections in this period. We note that the contribution from the symptomatic infectious individuals (*I*) is much higher than that from the asymptomatic infectious individuals (*E*) in shaping the disease risk in this period.

We plot the numerical curves of *S*, *E*, *I*, *R*, *M* and *C* in Figure 2(b)–(d). In particular, we observe that the value of the mitigation level *M* quickly increases in the first five days (March 28 to April 2) and then remains steady afterwards. The initial increase of *M* is obviously stimulated by the ascending phase of the epidemic. The stabilization afterwards is partly explained by the implementation of the state-wide Stay-at-Home order in Tennessee from April 1 to April 30. The Stay-at-Home order represents a high level of disease mitigation and outbreak management, and our model variable *M* reaches and stays at this high level after April 2, as shown in Figure 2(d). Meanwhile, we note that even under the Stay-at-Home regulation, essential businesses (such as grocery stores, pharmacies, convenience stores, mail and shipping services, home repair, and automotive sales and repair) were still open in the US, and this mitigation policy is doubtlessly weaker than a complete lock-down. This may explain that the value of *M* is stabilized at somewhere around 88% but not higher.

In parallel with the fast initial increase of the mitigation level *M*, there is a decrease of the economic development level *C* that starts from March 28 and continues until April 12, before it is finally stabilized. The economic development is negatively impacted by the disease control and management, and the reduction in the value of *C* accompanies the growth of *M*. Figure 2(d) shows that the rate of decrease for *C* (i.e., the downward slope) is smaller than the rate of increase for *M* (i.e., the upward slope), indicating some sort of resistance of the economic system in response to adverse conditions. The extended reduction of *C* beyond the increasing phase of *M* represents a delayed effect of the high mitigation level on the economic development. The curve of *C* is finally stabilized around 89.5%, consistent with the unemployment rate of 10.4% reported in Tennessee for the last week of April [47].

#### 3.1.2. Special cases

A major difference between our model (2.1) and a conventional epidemic model is the incorporation of two variables, *M* and *C*, related to economics. We now examine the impact of these two variables on the dynamics of system (2.1). To that end, we consider three special (and extreme) cases: *M* = 0, *C* = 0, and *M* = *C* = 0, with the same epidemic and economic datasets in Period 1 as described before.

For the first case, we remove the equation for *M* from system (2.1), and set *M* = 0 in the remaining equations for *S*, *E*, *I*, *R*, and *C*. Similarly, for the second case, we remove the equation for *C* from (2.1), and set *C* = 0 in the remaining equations for *S*, *E*, *I*, *R*, and *M*. For the third case, we remove the equations for *M* and *C* from (2.1), and set *M* = *C* = 0 in the remaining equations for *S*, *E*, *I*, and *R*. In each of these cases, we conduct data fitting for the corresponding reduced system and present the fitted parameter values in Table 3.

In order to compare the goodness-of-fit in different cases, we calculate the normalized root-mean-square error (NRMSE):

(3.2) $$\frac{\sqrt{n\sum_{k=1}^{n}{\left(Y_{k}-I_{k}\right)}^{2}}}{\sum_{k=1}^{n}Y_{k}}$$

where *n* is the number of days in this fitting period, *Y*_*k*_ is the reported number of active cases on the *k*-th day, and *I*_*k*_ is the numerical value of *I* on the *k*-th day. The values of the NRMSE for the three special cases *M* = 0, *C* = 0, *M* = *C* = 0, and the original case *M*, *C* ≠ 0, are found as 0.2036, 0.1287, 0.2218, and 0.1297, respectively. We see that, in particular, the first and third cases both lead to a lower quality of fitting than that associated with the original system (2.1).

**Figure 3.**
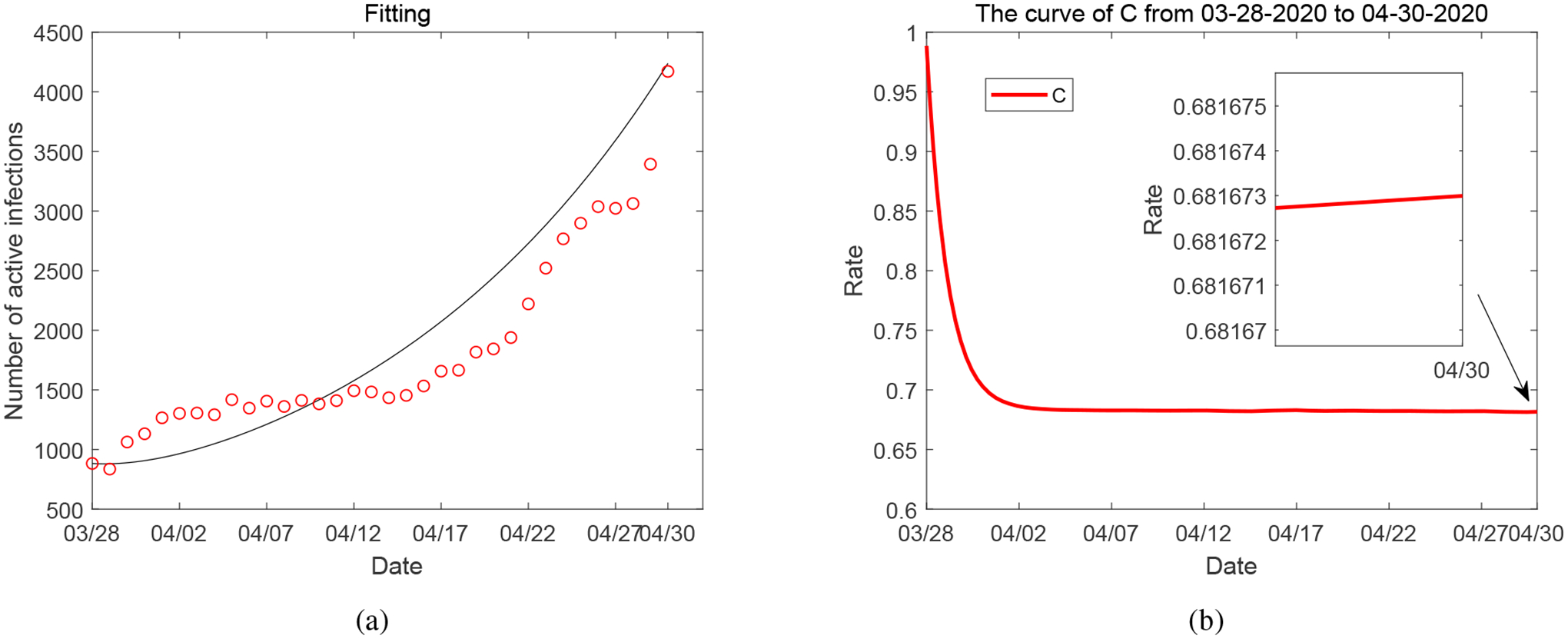
(a) Data fitting for active COVID-19 cases from March 28, 2020 to April 30, 2020 in Tennessee when *M* = 0. (b) The solutions of *C* from numerical simulation when *M* = 0.

We plot the result for the first case *M* = 0 in Figure 3. Comparing the parameter values in Table 3 to those in Table 2, we notice that the removal of the variable *M* leads to a significant over-estimate of the parameter *d*, the natural reduction rate of the economic development. This results in a rapid (and unrealistic) decrease of *C* to a level around 68%, as shown in Figure 3(b).

**Figure 4.**
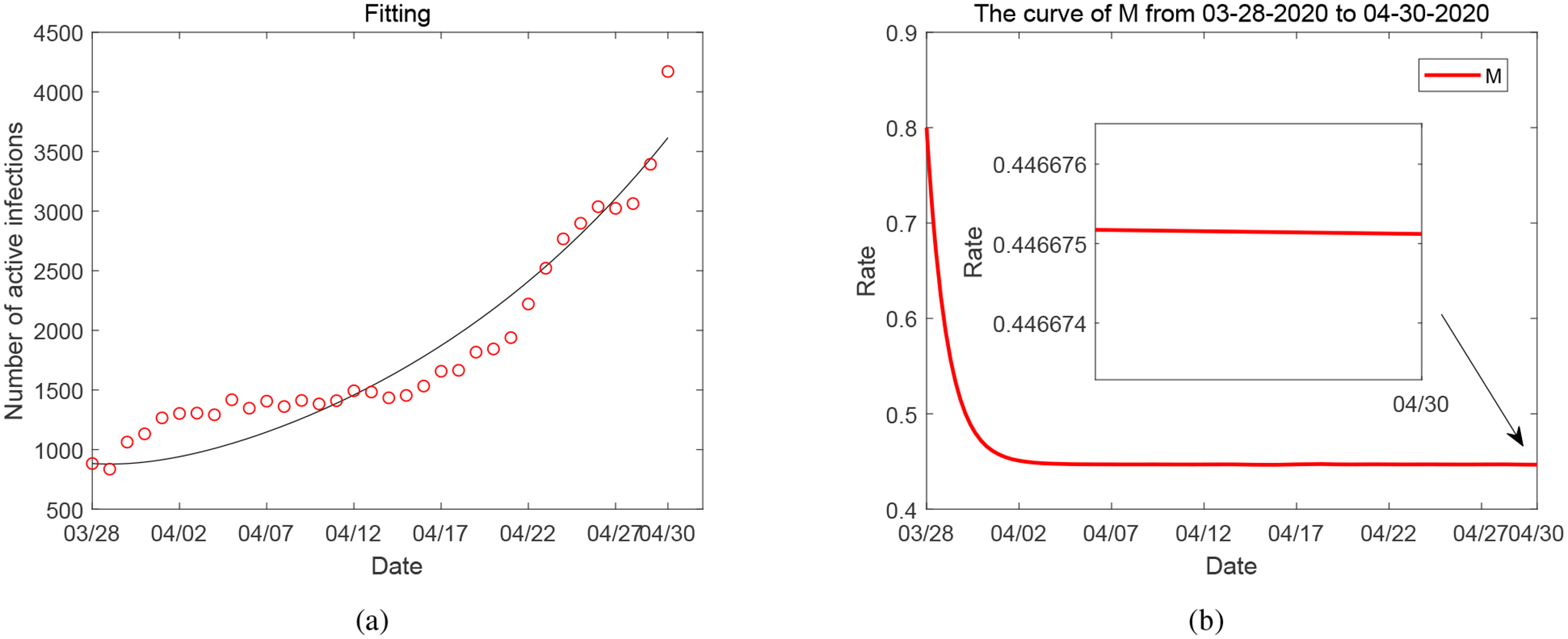
(a) Data fitting for active COVID-19 cases from March 28, 2020 to April 30, 2020 in Tennessee when *C* = 0. (b) The solutions of *M* from numerical simulation when *C* = 0.

Meanwhile, we plot the result for the second case *C* = 0 in Figure 4. Comparing Table 3 to Table 2, we observe that the removal of the variable *C* leads to an over-estimate of the parameter *p*, the natural reduction rate of the mitigation level, and an under-estimate of the parameter *b*, the implementation rate of the mitigation. These similarly result in a rapid (and unrealistic) decrease of *M* to a level around 45%, as shown in Figure 4(b).

**Figure 5.**
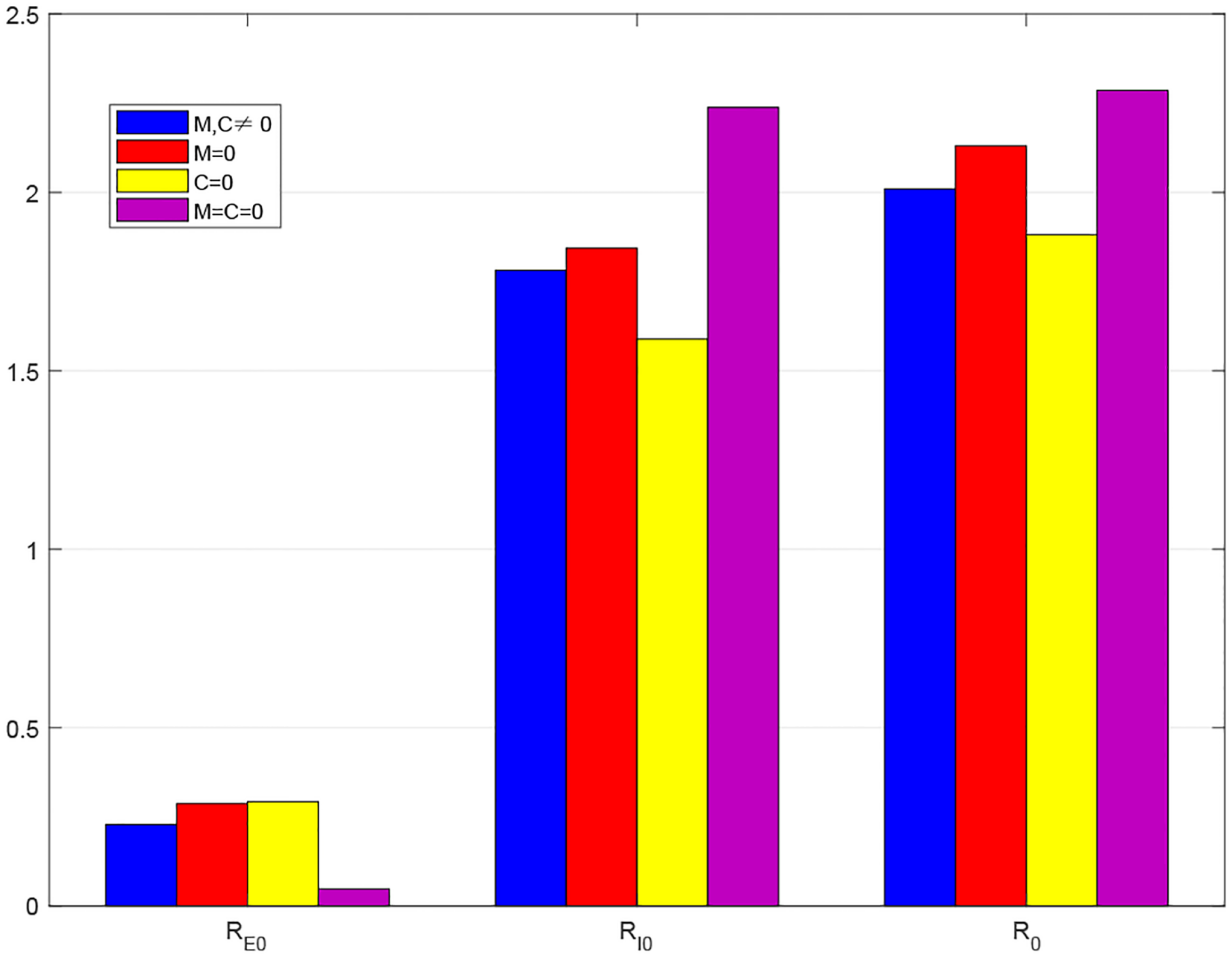
Comparison of the reproduction numbers in four cases: *M*, *C* ≠ 0, *M* = 0, *C* = 0, and *M* = *C* = 0, based on data in Period 1.

Figure 5 displays a histogram to compare the reproduction numbers in the three special cases (*M* = 0, *C* = 0, *M* = *C* = 0) and the original case with nonzero *M* and *C* for Period 1. In each case, we observe that the component $R_{I0}$ is much higher than the other component $R_{E0}$, showing that symptomatic infections contribute to most part of the disease risk in this period. Overall, the basic reproduction number $R_{0}$ takes a lower value when *C* = 0 than that in the original case *M*, *C* ≠ 0, indicating a lower disease risk in the extreme scenario where economic development is completely disregarded. On the other hand, $R_{0}$ takes a higher value when *M* = 0 and *M* = *C* = 0 than that in the original case, indicating a higher disease risk in the extreme scenario where mitigation strategies are totally abandoned.

#### 3.1.3. Sensitivity analysis

To quantify the influence of model parameters on disease dynamics, we conduct a sensitivity analysis [53] for the parameters in system (2.1) using the data in Period 1. Table 4 displays the results for the relative sensitivity of the basic reproduction numbers, $R_{10}$ in Period 1, in terms of model parameters. The relative sensitivity of $R_{10}$ with respect to *β*_*E*_, for example, is computed by

(3.3) $$\frac{\partial R_{10}}{\partial \beta_{E}}\cdot \frac{\beta_{E}}{R_{10}}=\frac{\beta_{E}}{\beta_{E}+\frac{\alpha \beta_{I}}{\omega +\gamma +\mu }},$$

where Eq (2.2) has been used. Similar calculation applies to other parameters.

**Figure 6.**
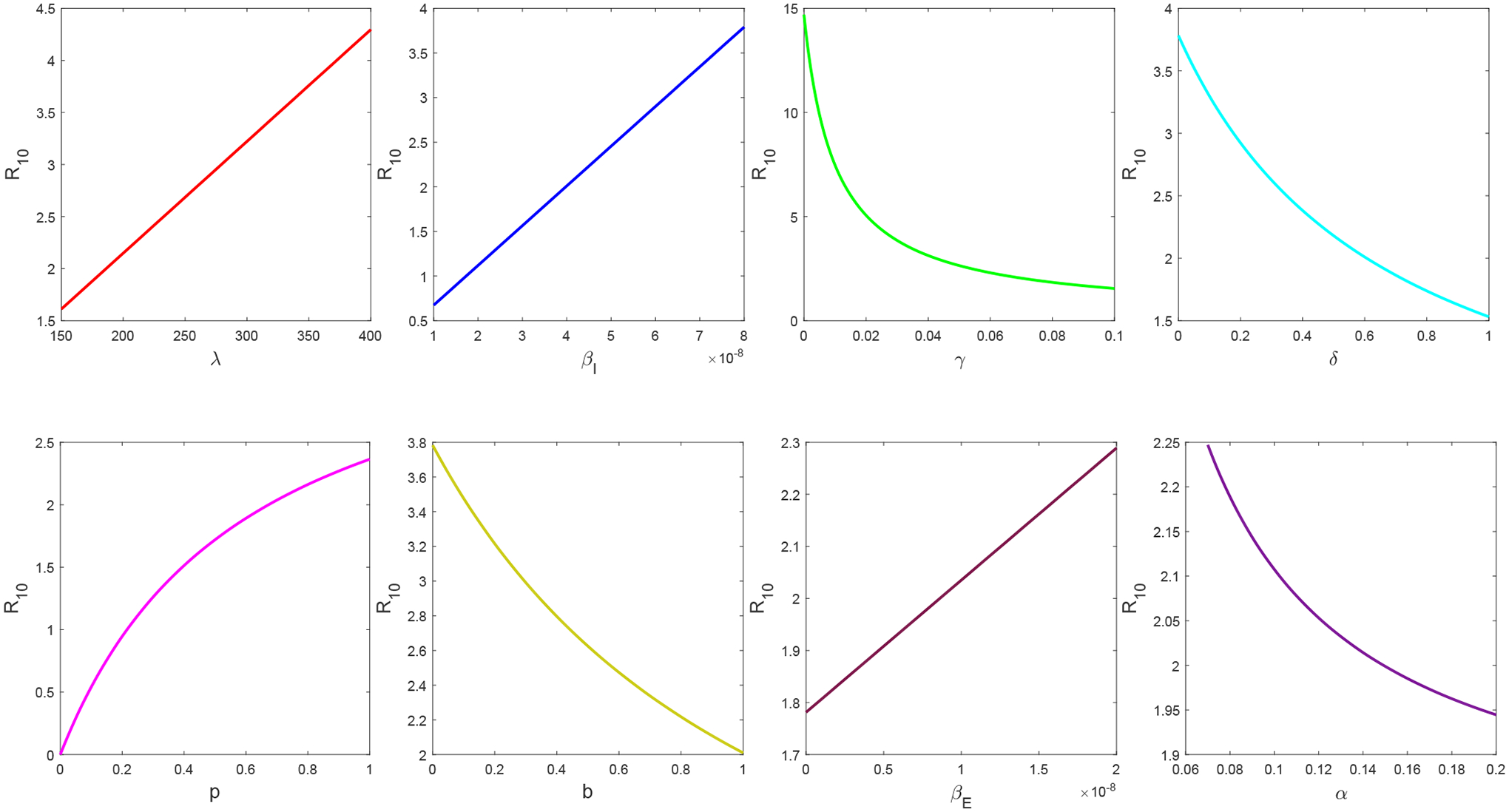
Variations of $R_{10}$, the basic reproduction number in Period 1, in terms of the eight most sensitive parameters.

Figure 6 illustrates the variations of $R_{10}$ in terms of the eight most sensitive parameters ranked in Table 4. Practically, among these eight parameters, at least five could be controlled through public health management: the transmission rates *β*_*I*_ and *β*_*E*_, the mitigation influx rate *δ*, the mitigation decline rate *p*, and the mitigation implementation rate *b*. In particular, social distancing, mask wearing, and isolation of infected individuals would bring down the transmission rates *β*_*I*_ and *β*_*E*_ and could be most effective in reducing the disease risk. Meanwhile, strengthening epidemic management would increase the mitigation influx rate *δ* and implementation rate *b*, and promoting public awareness of the infection would weaken the mitigation decline rate *p*. These could also effectively reduce the value of the basic reproduction number. Additionally, improved treatment modalities, such as discovery of more effective therapeutic drugs for SARS-CoV-2, may be able to enlarge the recovery rate *γ* (i.e., decrease the length of the average recovery period for COVID-19 patients) and help to reduce the overall risk of the epidemic.

**Figure 7.**
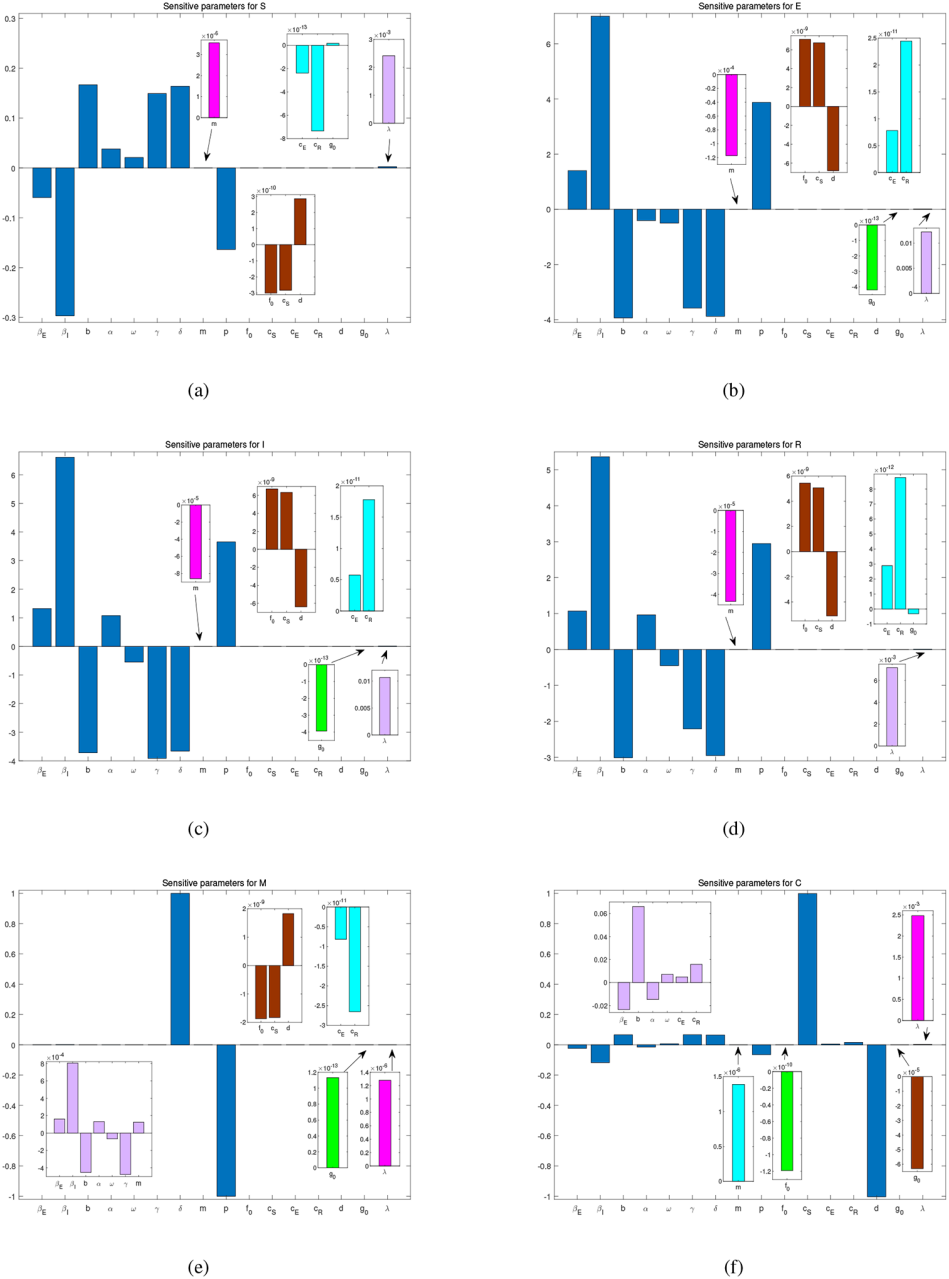
Sensitivity of model parameters for the six state variables in system (2.1): (a) *S*; (b) *E*; (c) *I*; (d) *R*; (e) *M*; and (f) *C*.

Using the method described in [33], we have also computed the relative sensitivity of all the six state variables in model (2.1) with respect to these parameters, and the results are presented in Figure 7. In particular, the relative sensitivity of a state variable, *Y*, with respect to *β*_*E*_ is given by $\frac{\partial Y}{\partial \beta_{E}}\cdot \frac{\beta_{E}}{Y}$. To obtain the partial derivative $\frac{\partial Y}{\partial \beta_{E}}$, we differentiate it with respect to *t* to obtain

(3.4) $$\frac{\partial }{\partial t}\left(\frac{\partial Y}{\partial \beta_{E}}\right)=\frac{\partial }{\partial \beta_{E}}\left(\frac{\partial Y}{\partial t}\right),\text{ where }Y=S,E,I,R,M,C\text{. }$$

We then numerically solve for each $\frac{\partial Y}{\partial \beta_{E}}$ by connecting Eqs (2.1) and (3.4). The sensitivity computation for other parameters is similar.

We observe from Figure 7 that the variables *S*, *E*, *I* and *R* are all very sensitive to the parameters *β*_*I*_, *b*, *γ* and *δ*. Meanwhile, all the state variables except *C* have a high level of sensitivity with respect to *p*. Additionally, the variable *M* is highly sensitive to *δ*. On the other hand, *C* is highly sensitive to *d*, the natural reduction rate of economic development, and *c*_*S*_, the economic contribution rate from the susceptible individuals that would account for the major labor supply. These parameters, except *d* and *c*_*S*_, are also ranked among the most sensitive parameters for the basic reproduction number in Table 4. The fact that the variable *C* and the basic reproduction number $R_{10}$ have different sensitivity dependence on parameters is a reflection of the different focuses between economic development and public health mitigation.

#### 3.1.4. Simulated scenarios

Our sensitivity analysis in Section 3.1.3 has identified seven parameters: *β*_*I*_, *b*, *γ*, *δ*, *p*, *d*, and *c*_*S*_, which are highly sensitive for the model outcome. We now conduct a detailed simulation to quantify the change of the model variables when the values of these parameters vary. We do not consider here the population influx rate *λ* since it is a demographic characteristic that is largely independent of public health management and short-term economic development. We focus on the three state variables *I*, *M* and *C* in the discussion below. For comparison, we refer to the parameter values provided in Table 2 for Period 1 as their base values, and the simulation result presented in Figure 2 as the base scenario.

##### Variation of β_I_.

**Figure 8.**
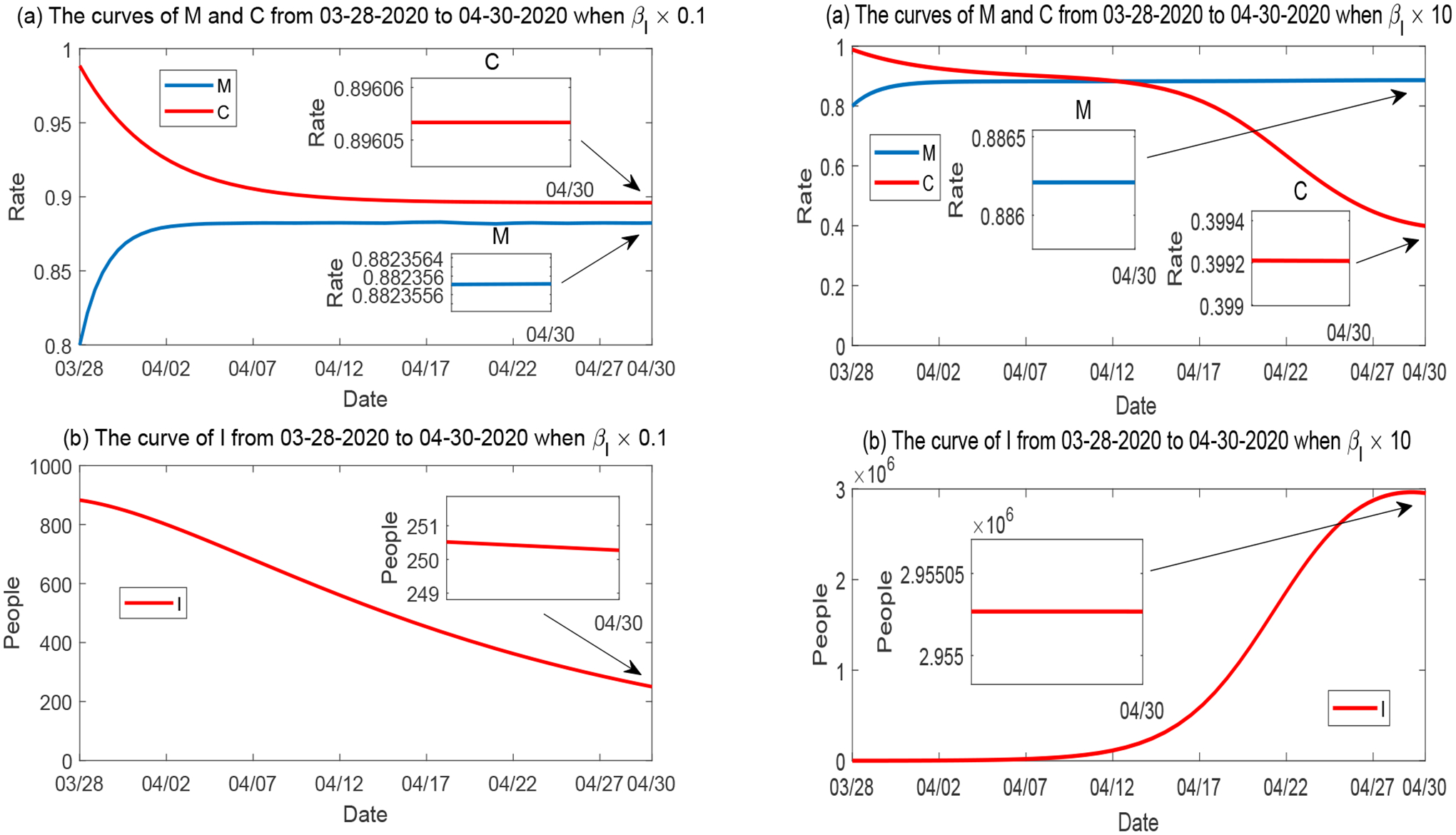
Numerical solutions of *M*, *C* and *I* when the symptomatic transmission rate *β*_*I*_ varies, while all other parameters are fixed. Left panel: *β*_*I*_ is reduced to 10%; Right panel: *β*_*I*_ is increased by 10 times.

We first examine the impact of the symptomatic transmission rate *β*_*I*_. We consider a scenario where *β*_*I*_ is reduced to 10% of its base value given in Table 2, and another scenario where *β*_*I*_ is increased by 10 times of its base value. Figure 8 displays the curves of *M*, *C* and *I* in these two hypothetical scenarios, where all the other parameters are fixed at values given in Table 2. The left panel of Figure 8 shows that, although the reduction of *β*_*I*_ does not have much impact on *M* and *C*, it substantially changes the behavior of *I*. As a result, the curve of *I* keeps decreasing throughout Period 1 due to the significant reduction of disease transmission. On the other hand, the right panes shows that the 10-fold increase of *β*_*I*_ leads to a dramatic increase in the number of infections, up to 3 × 10^6^ which is more than 43% of the total population in Tennessee. Consequently, this causes a sharp decrease in the economic development *C*, down to a level around 40%, due to the significant loss of labor supply.

##### Variation of γ.

**Figure 9.**
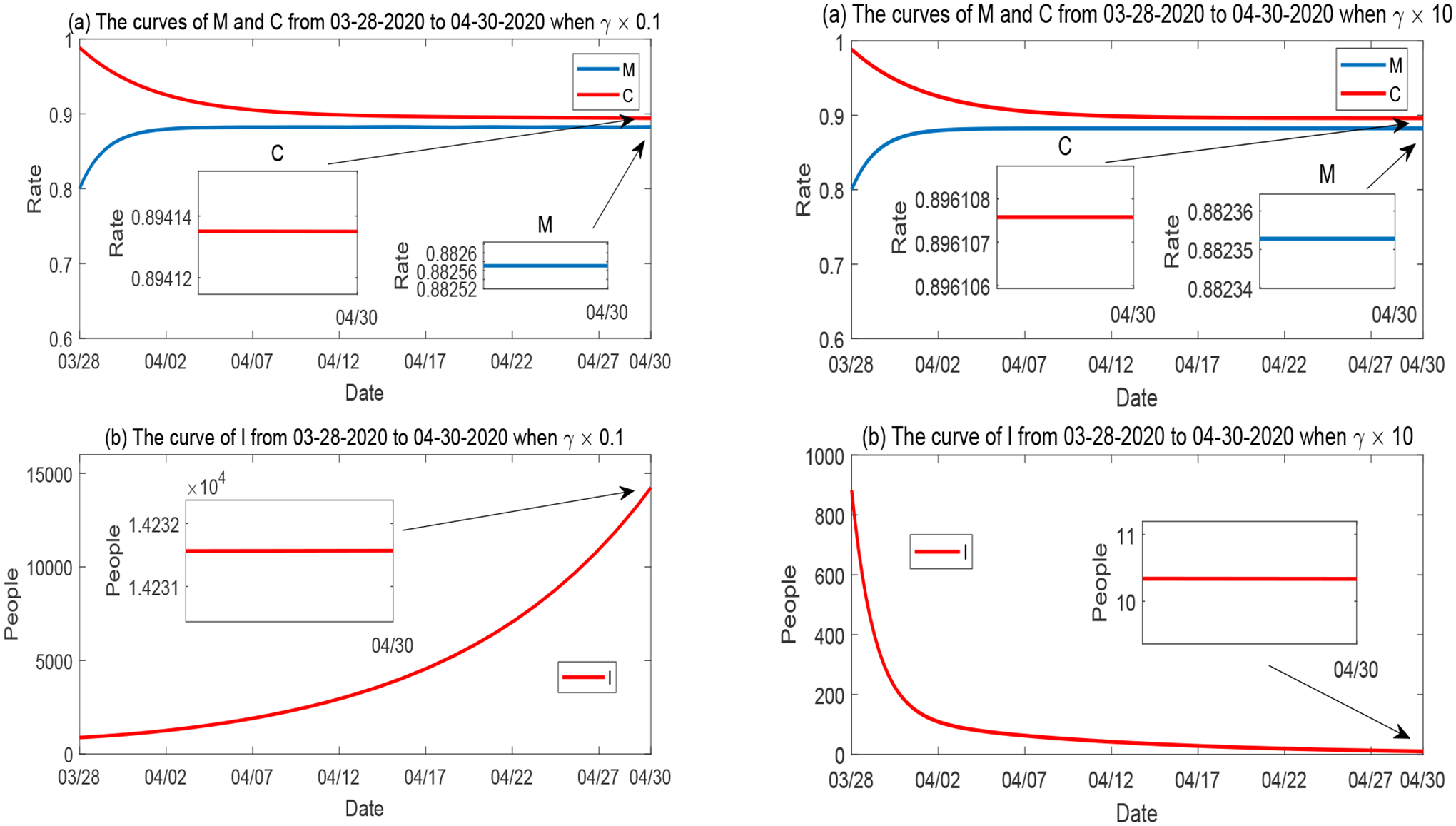
Numerical solutions of *M*, *C* and *I* when the recovery rate *γ* varies, while all other parameters are fixed. Left panel: *γ* is reduced to 10%; Right panel: *γ* is increased by 10 times.

Next, we consider the impact of the recovery rate *γ*. Figure 9 shows the simulation results when *γ* is hypothetically reduced to 10% (left panel), and hypothetically increased by 10 times (right panel), in reference to its base value provided in Table 2. All other parameters are fixed. As can be naturally expected, the lower value of *γ* leads to an increase of the number of active infections due to the prolonged disease recovery, while the higher value of *γ* leads to a reduction of the number of active infections due to the shortened recovery period. In contrast, the variation of *γ* has little influence on *M* and *C*, indicating their low sensitivity on the recovery rate, which is consistent with the sensitivity results presented in Figure 7(e),(f).

##### Variation of δ.

**Figure 10.**
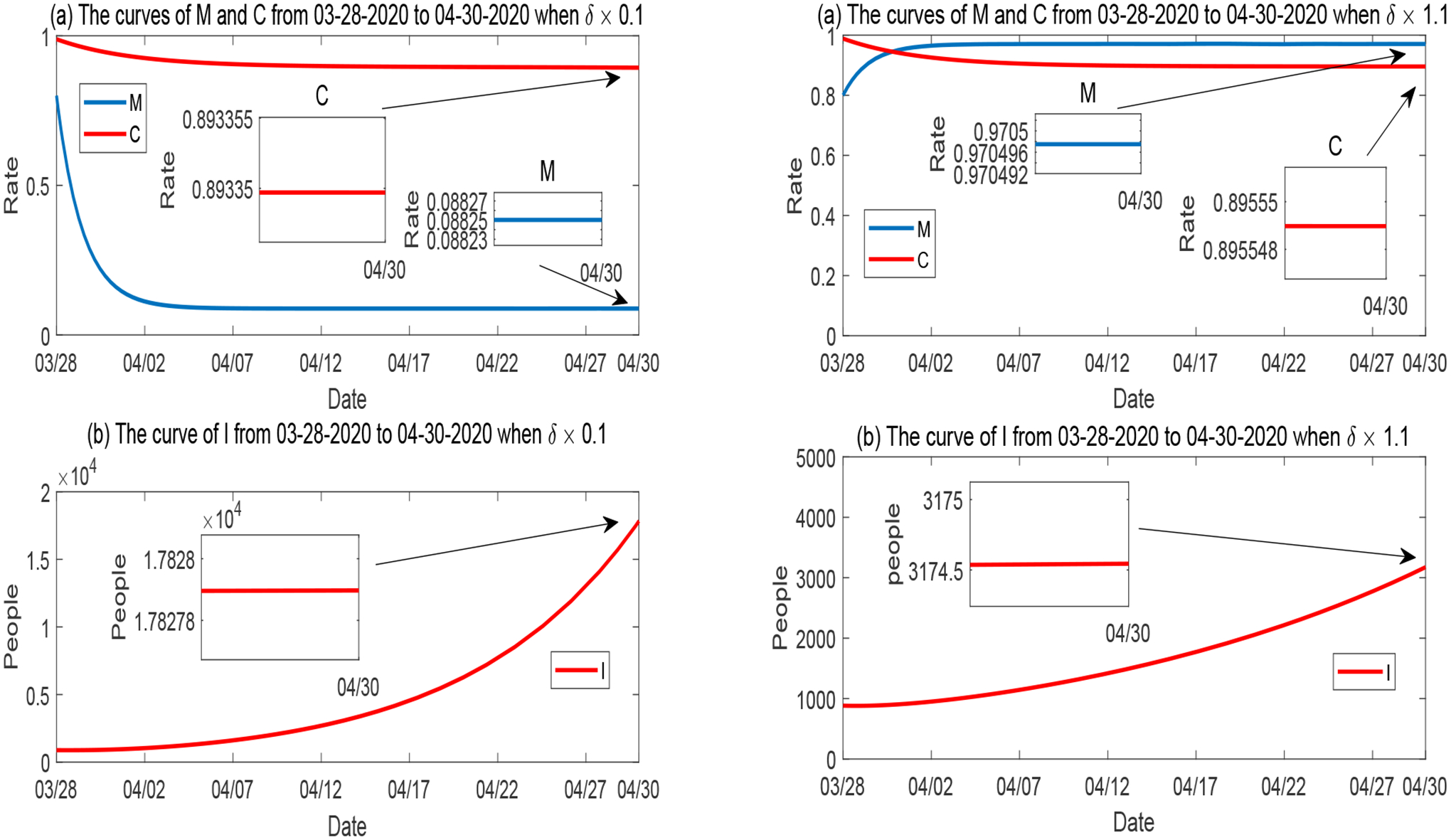
Numerical solutions of *M*, *C* and *I* when the influx rate of mitigation *δ* varies, while all other parameters are fixed. Left panel: *δ* is reduced to 10%; Right panel: *δ* is increased to 110%.

We plot the simulation result with respect to the variation of the mitigation influx rate *δ* in Figure 10. As observed in Figure 7, *M* and *I* are positively and negatively correlated to *δ*, respectively, and both *M* and *I* are highly sensitive to *δ*, whereas *C* has a low sensitivity level for *δ*. The left panel of Figure 10 demonstrates that when *δ* is reduced to 10% of its base value, *M* rapidly decreases to a level around 9%, and *I* quickly increases to a number about 5 times of the base scenario in Figure 2(c). The right panel illustrates that when *δ* is just increased by 10% from its base value, *M* would rise to a very high level (around 97%) and *I* would be at a level lower than the base scenario in Figure 2(c).

##### Variation of c_S_.

**Figure 11.**
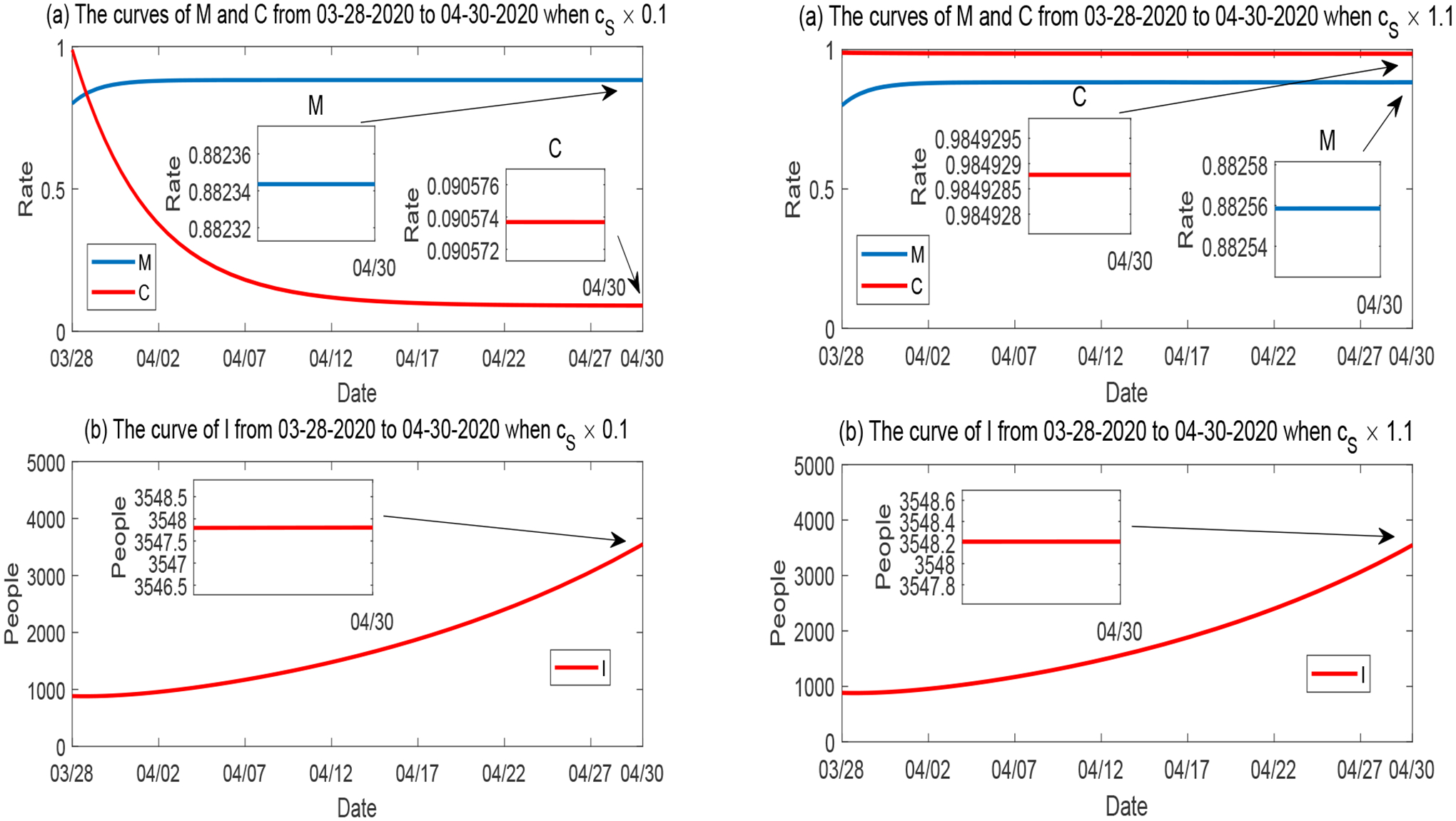
Numerical solutions of *M*, *C* and *I* when the labor contribution rate *c*_*S*_ varies, while all other parameters are fixed. Left panel: *c*_*S*_ is reduced to 10%; Right panel: *c*_*S*_ is increased to 110%.

We learn from Figure 7(f) that *C* is positively correlated and highly sensitive to the labor contribution rate *c*_*S*_. Figure 11 illustrates the two hypothetical scenarios when *c*_*S*_ is reduced to 10% of its base value (left panel), where *C* dramatically decreases to a level around 9%, and when *c*_*S*_ is increased to 110% of its base value (right panel), where *C* reaches a very high level (around 98%). There is little change in the *M* and *I* curves when *c*_*S*_ varies.

##### Variations of p, d and b.

**Figure 12.**
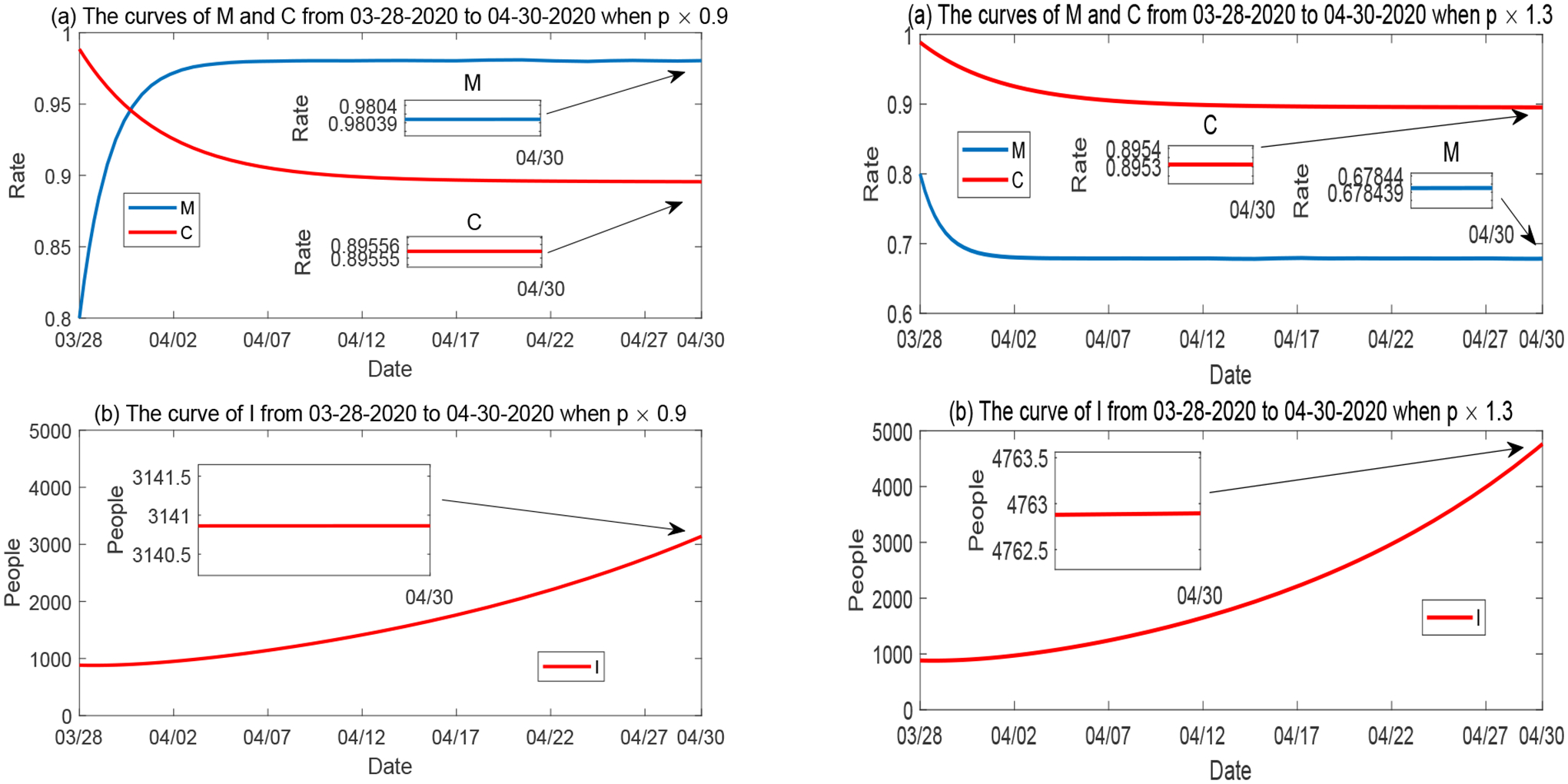
Numerical solutions of *M*, *C* and *I* when the natural reduction rate of mitigation *p* varies, while all other parameters are fixed. Left panel: *p* is reduced to 90%; Right panel: *p* is increased by 1.3 times.

**Figure 13.**
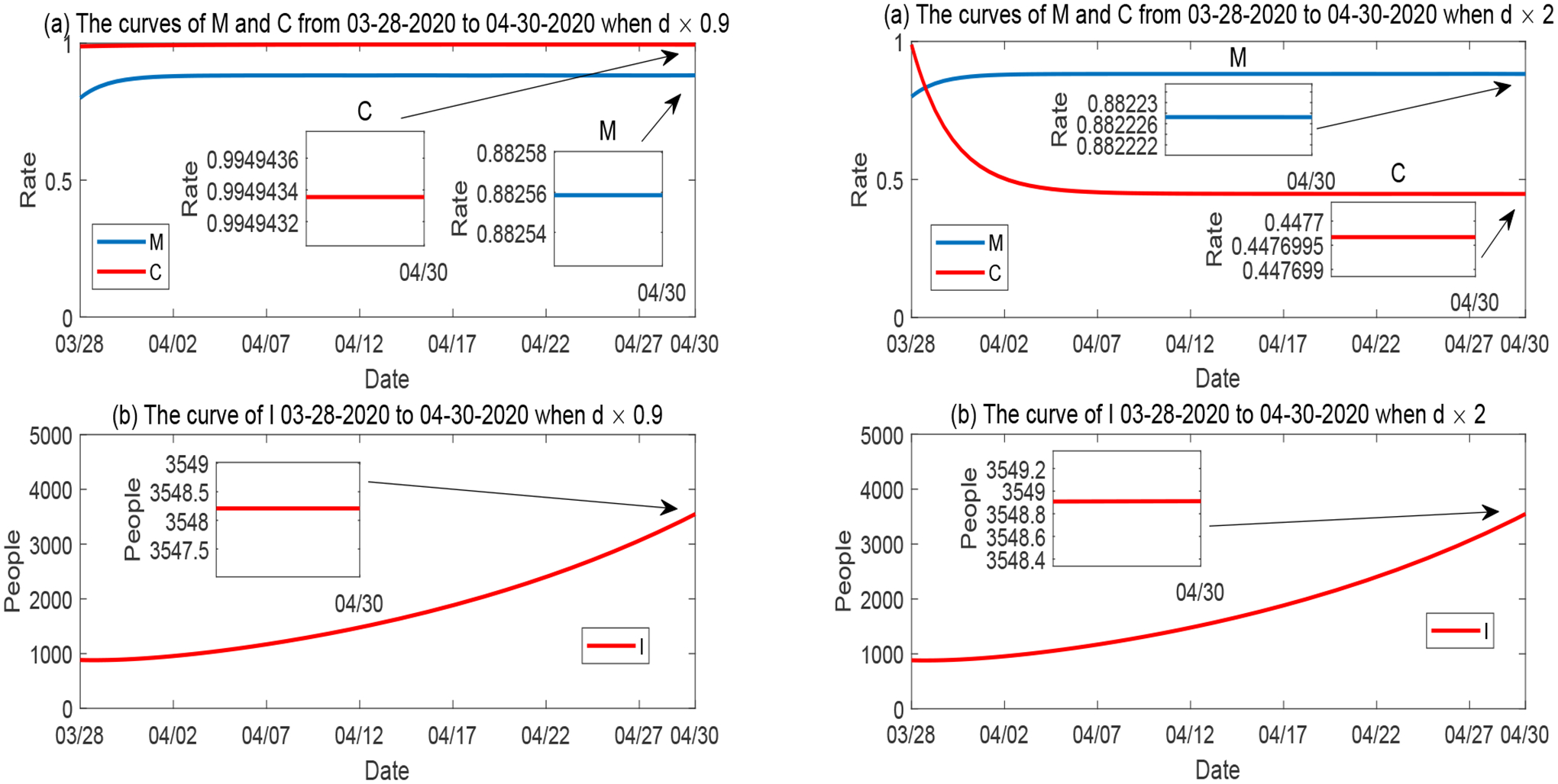
Numerical solutions of *M*, *C* and *I* when the natural reduction rate of economic development *d* varies, while all other parameters are fixed. Left panel: *d* is reduced to 90%; Right panel: *d* is increased by 2 times.

**Figure 14.**
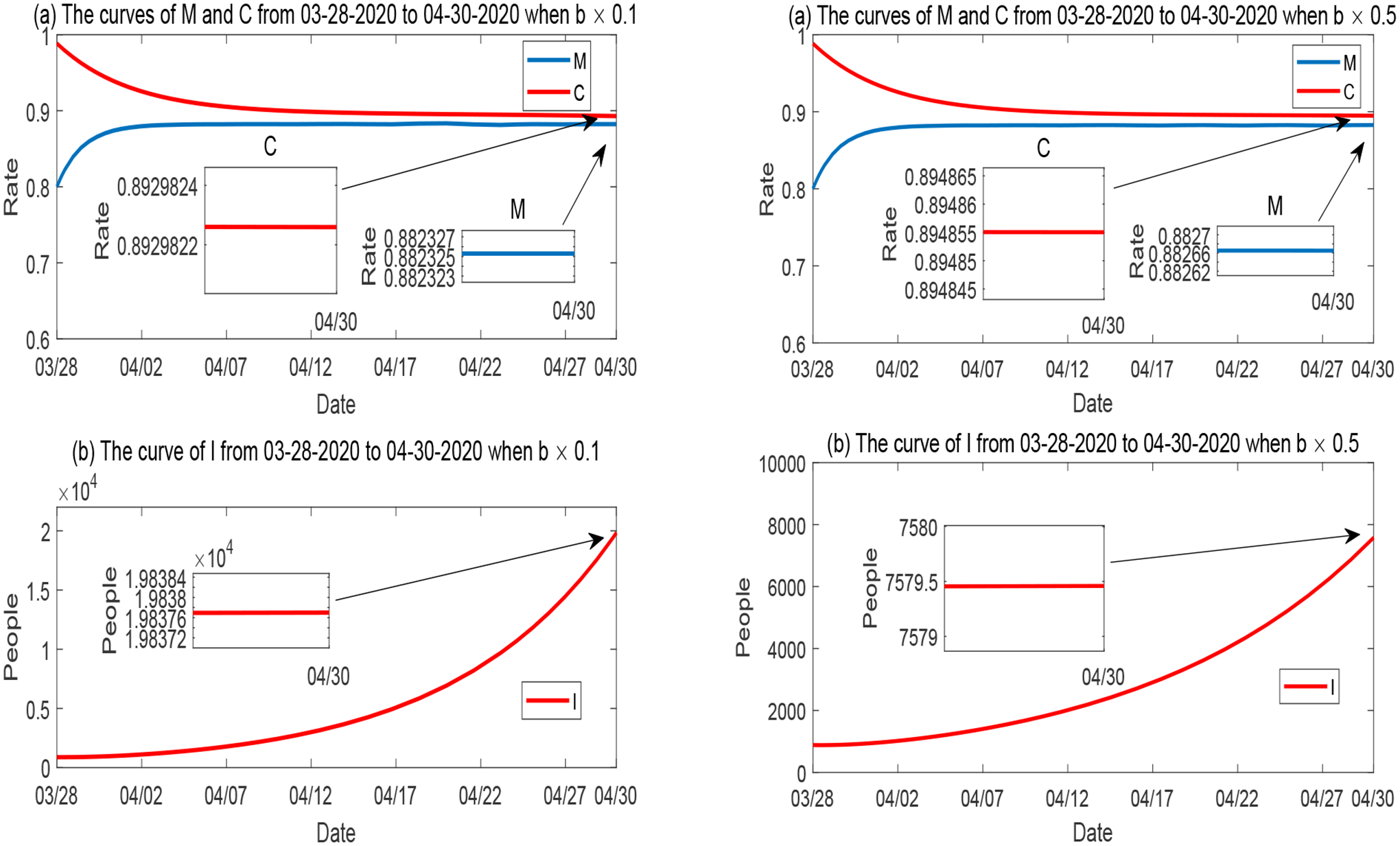
Numerical solutions of *M*, *C* and *I* when the mitigation implementation rate *b* varies, while all other parameters are fixed. Left panel: *b* is reduced to 10%; Right panel: *b* is reduced to 50%.

Furthermore, we plot the simulation results for the variation of *p* (the natural reduction rate of disease mitigation) in Figure 12, for the variation of *d* (the natural reduction rate of economic development) in Figure 13, and for the variation of *b* (the mitigation implementation rate) in Figure 14. The behaviors of *M*, *C* and *I* with respect to the variations of these parameters are consistent with the sensitivity results in Figure 7.

**Figure 15.**
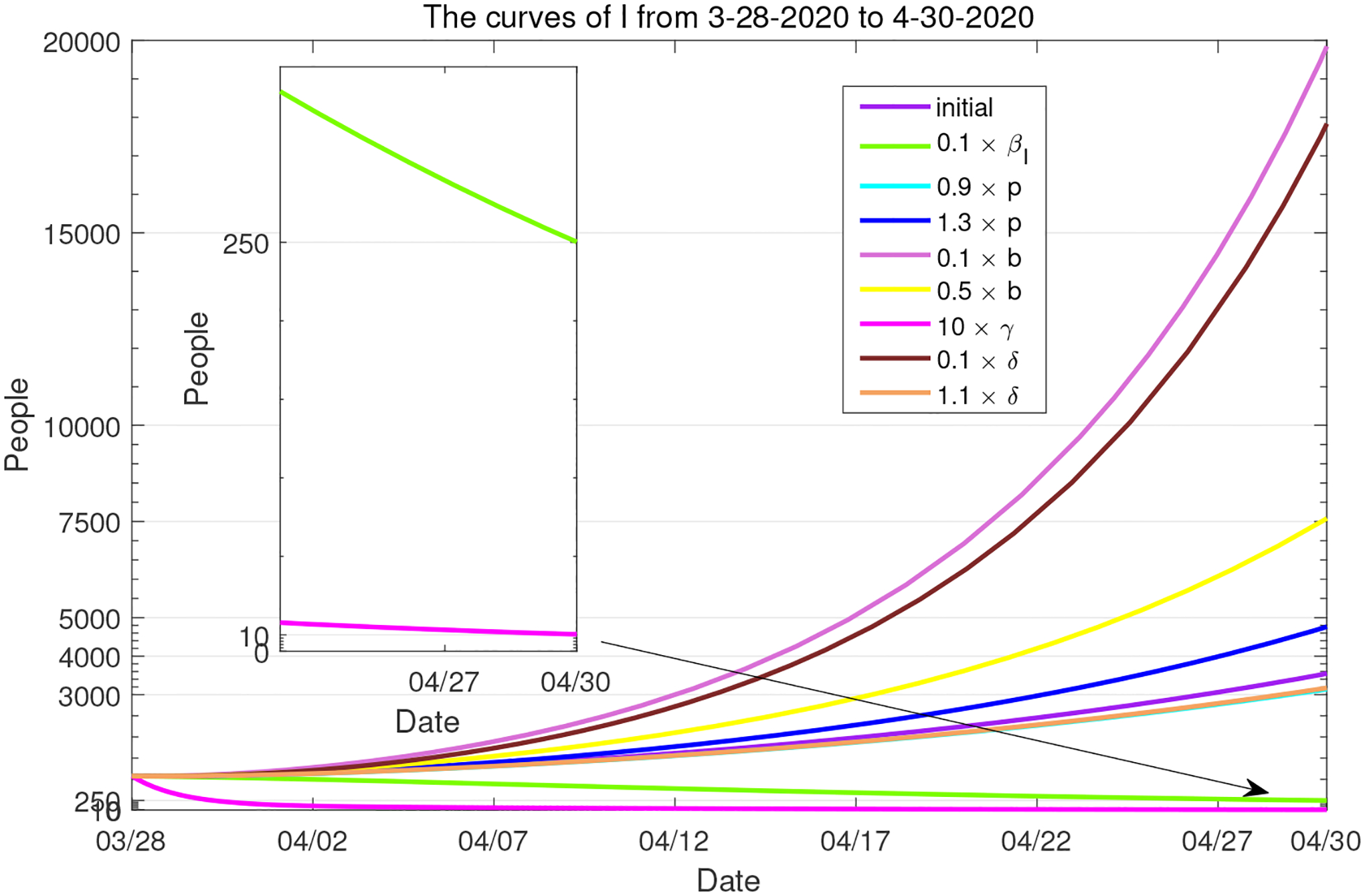
Combined numerical solutions of *I* in Period 1 with respect to several parameter variations. The purple line represents the base scenario.

**Figure 16.**
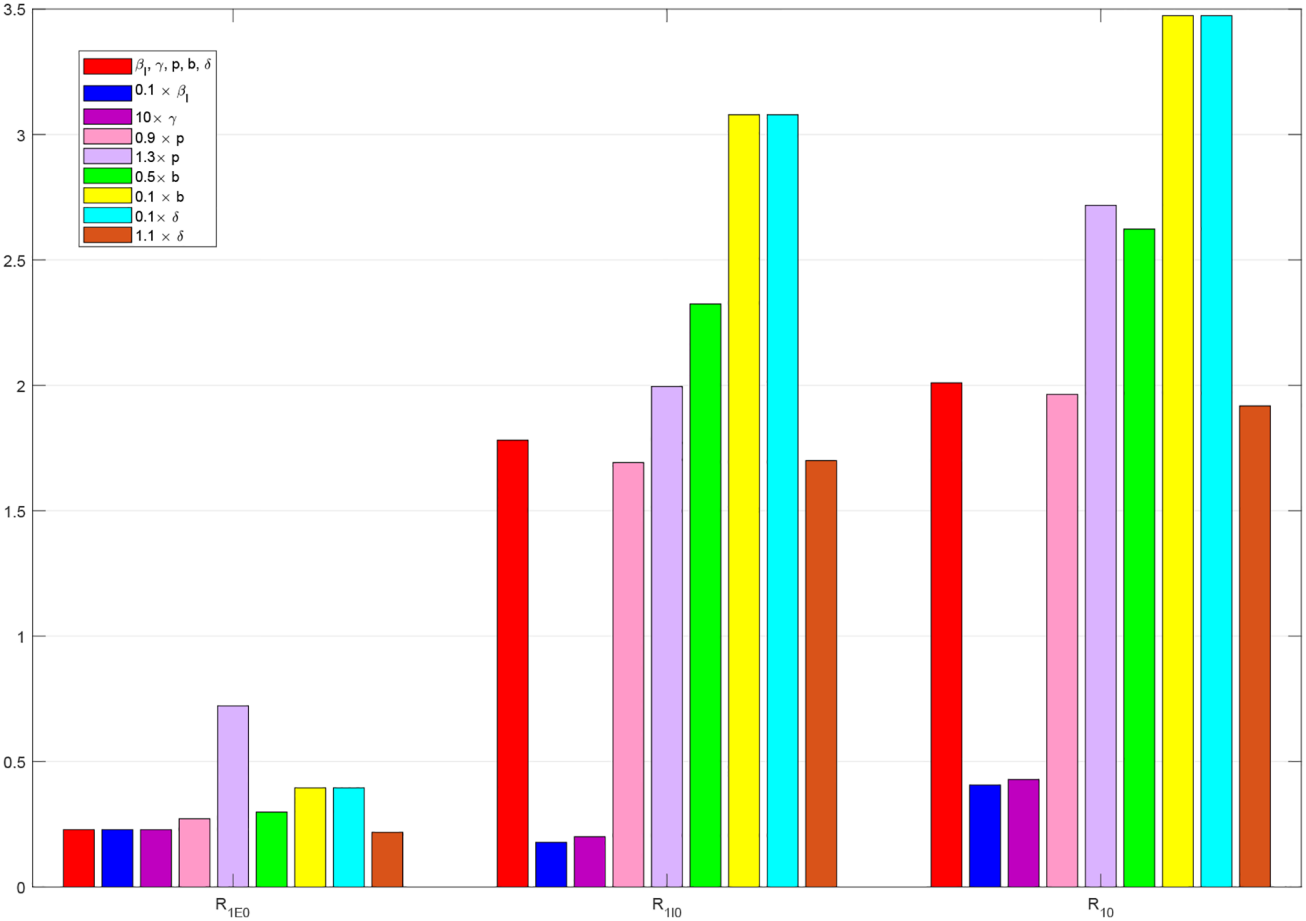
Changes of the reproduction numbers in Period 1 with respect to several parameter variations. The red bars represent the base scenario.

Additionally, we have combined the solution curves of *I* in most of these simulated scenarios and presented the results in Figure 15, so that we may easily observe the quantitative impacts of these parameter variations on the number of active infections in Period 1. Among these presented scenarios, the reduction of *I* is most significant with the (hypothetical) increase of the recovery rate *γ* and the decrease of the transmission rate *β*_*I*_. Note that a few off-the-scale scenarios are not included in this plot. Moreover, a histogram for the changes of the reproduction numbers under most of these parameter variations is presented in Figure 16, which provides a quantitative demonstration of the sensitivity prediction in Table 4. Consistent with the result in Figure 15, the decrease of *β*_*I*_ and increase of *γ* appear most effective in reducing the value of the basic reproduction number.

### 3.2. Fitting and simulation in Periods 2–5

**Figure 17.**
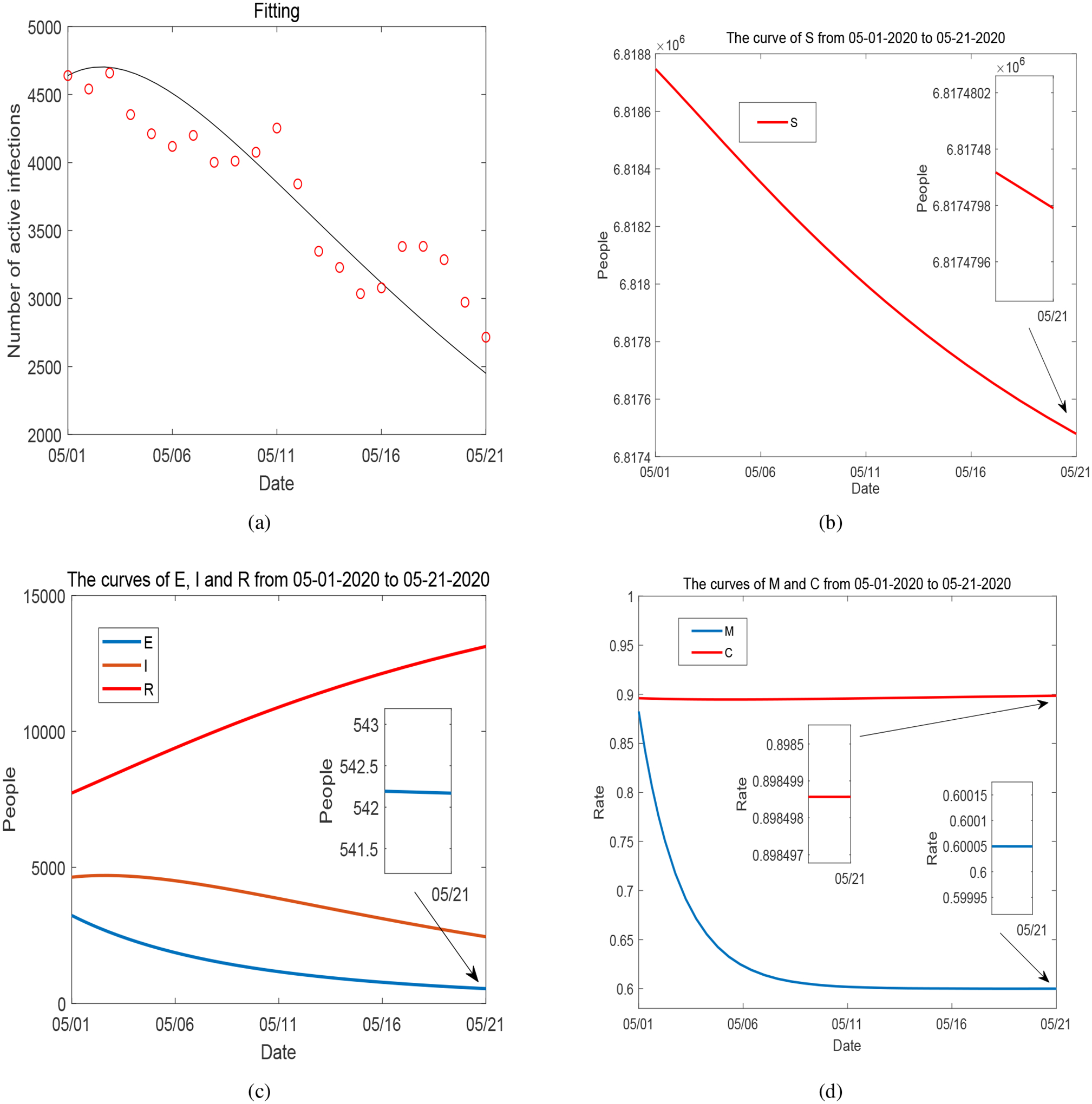
(a) Data fitting for active COVID-19 cases in Tennessee during Period 2 (from May 1, 2020 to May 21, 2020): circles represent the reported data and solid line represents the fitting result. (b)–(d): Curves of *S*, *E*, *I*, *R*, *M* and *C* from numerical simulation.

**Figure 18.**
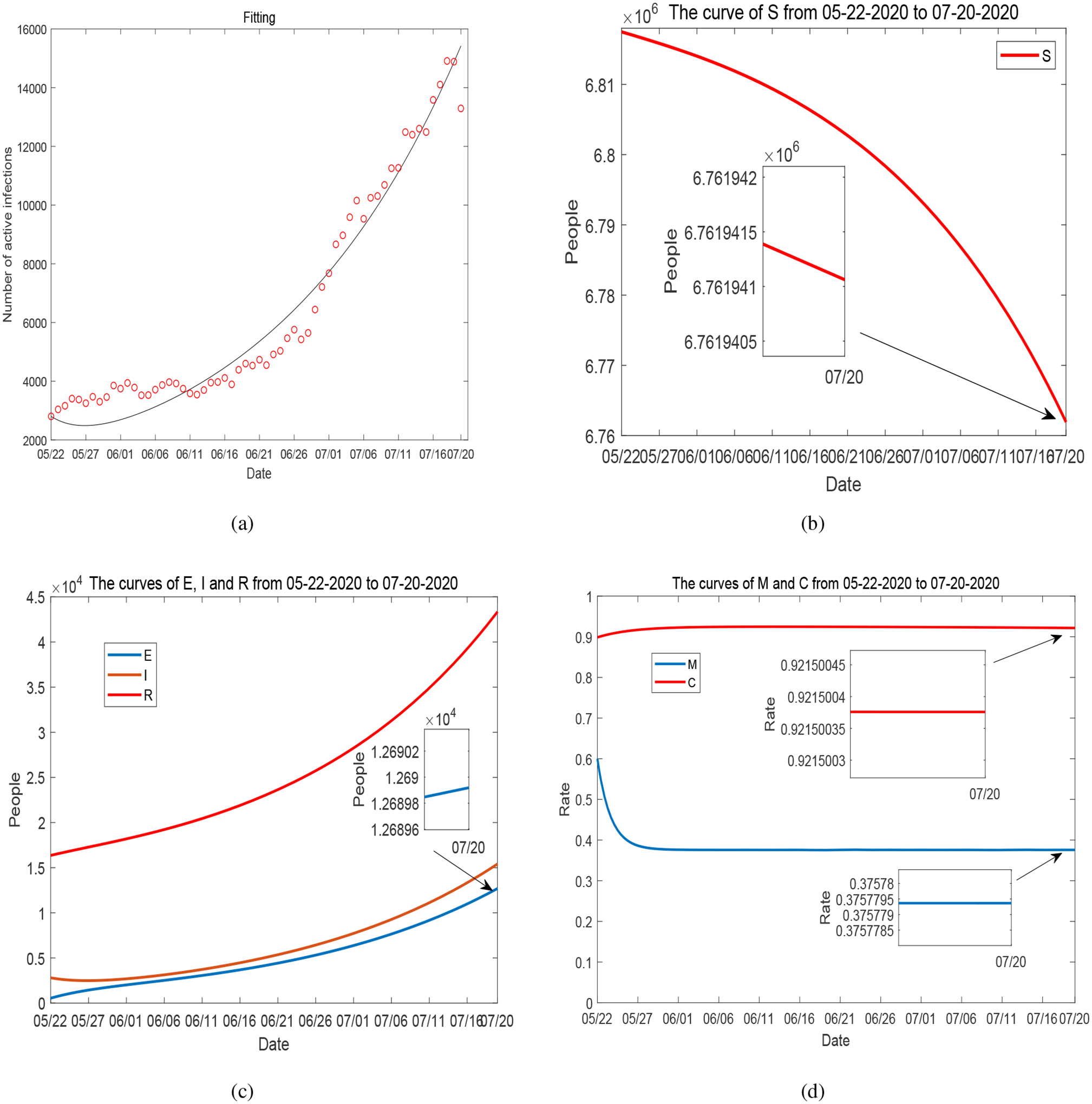
(a) Data fitting for active COVID-19 cases in Tennessee during Period 3 (from May 22, 2020 to July 20, 2020): circles represent the reported data and solid line represents the fitting result. (b)–(d): Curves of *S*, *E*, *I*, *R*, *M* and *C* from numerical simulation.

**Figure 19.**
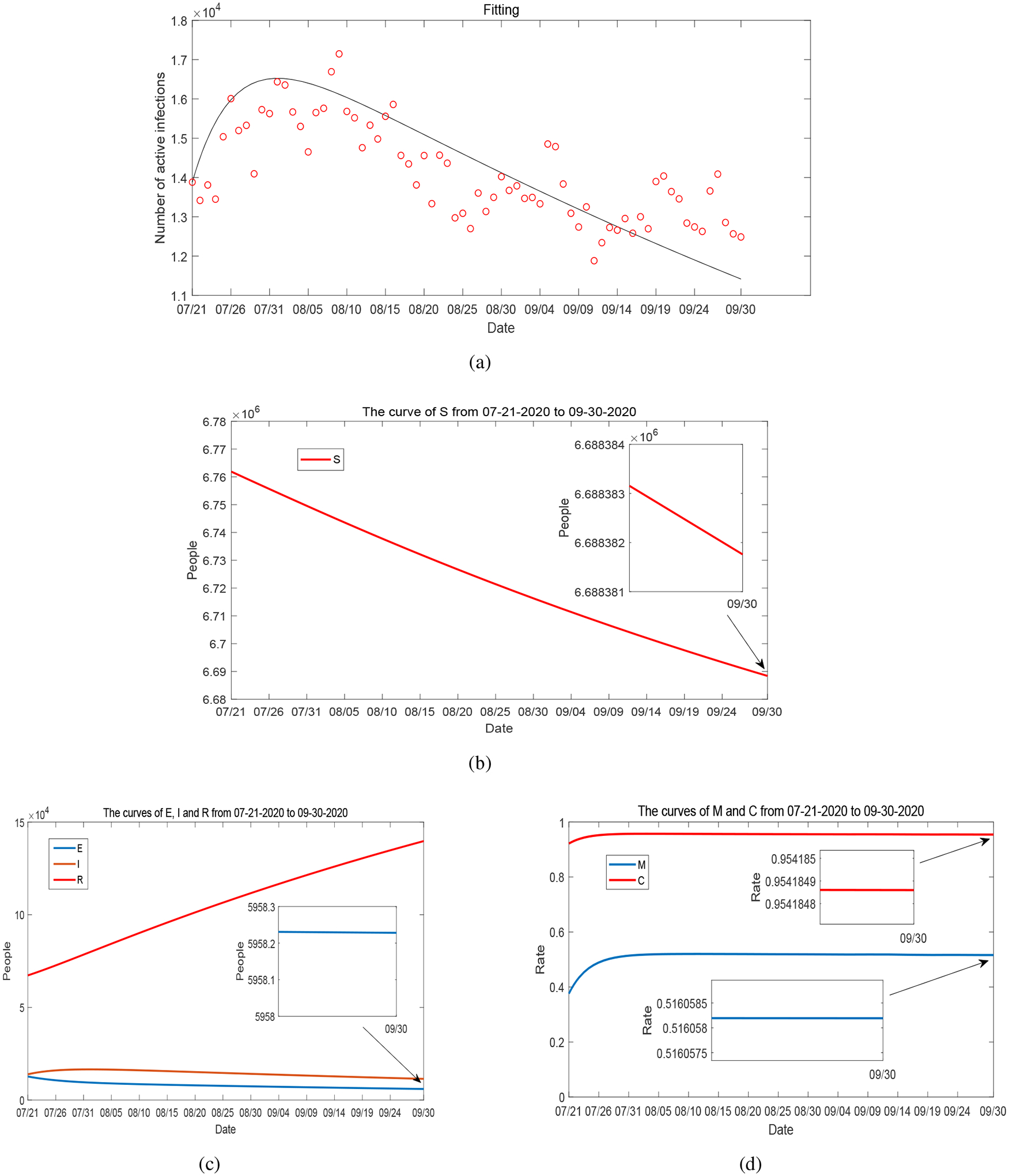
(a) Data fitting for active COVID-19 cases in Tennessee during Period 4 (from July 21, 2020 to September 30, 2020): circles represent the reported data and solid line represents the fitting result. (b)–(d): Curves of *S*, *E*, *I*, *R*, *M* and *C* from numerical simulation.

**Figure 20.**
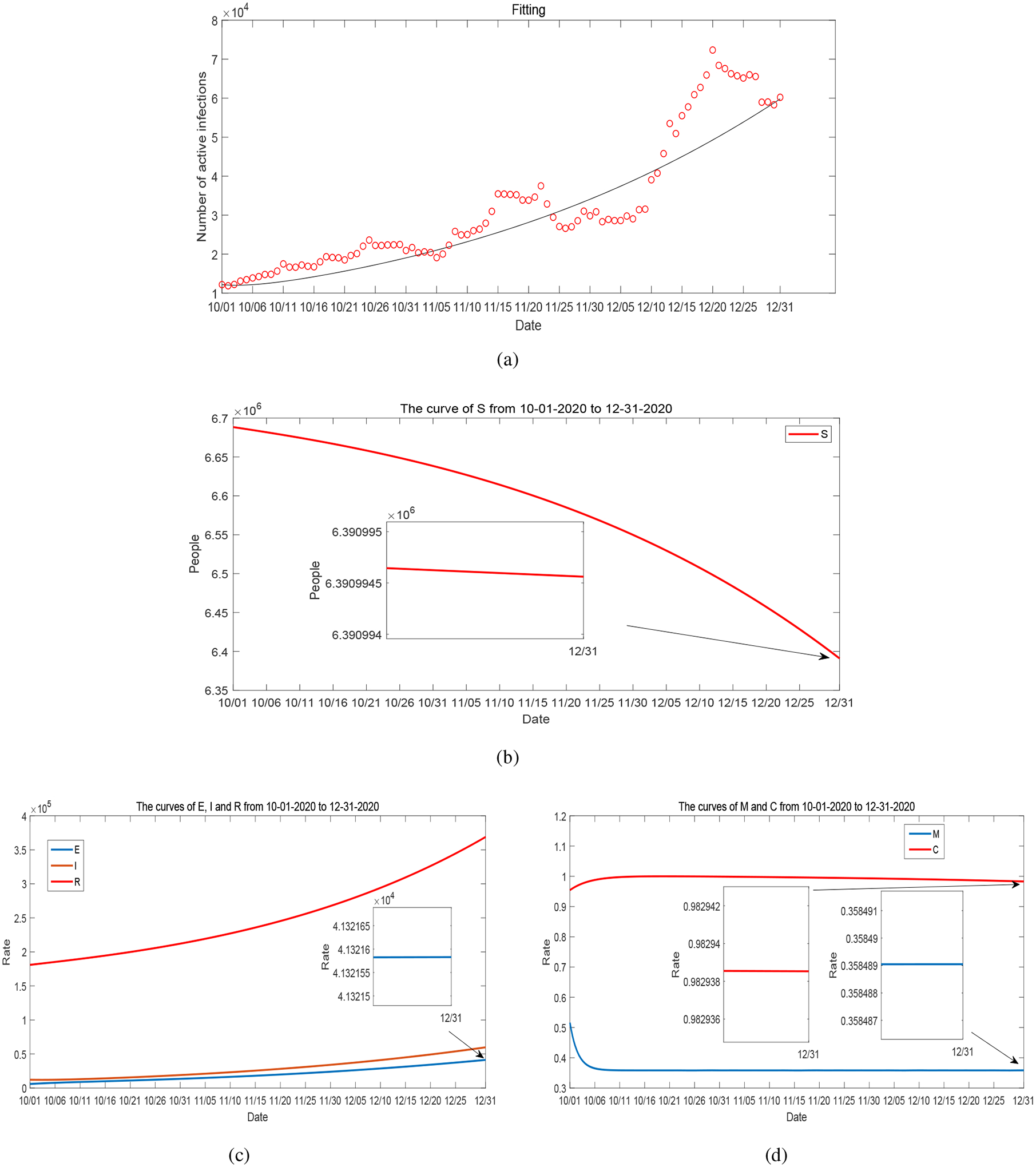
(a) Data fitting for active COVID-19 cases in Tennessee during Period 5 (from October 1, 2020 to December 31, 2020): circles represent the reported data and solid line represents the fitting result. (b)–(d): Curves of *S*, *E*, *I*, *R*, *M* and *C* from numerical simulation.

We have presented and discussed our detailed simulation results for Period 1 in Section 3.1. For completeness, we summarize the data fitting and numerical results for Periods 2–5 in Figures 17–20. These four periods represent the time from May 1, 2020 to December 31, 2020. The values of fitted parameters for each period are listed in Table 2. We observe several ups and downs for the curves of *I*, *M* and *C* throughout these periods, in accordance with the evolution of the infected cases and unemployment rates reported in Tennessee and the varied disease risk levels at different times. Based on the fitted parameters, the basic reproduction number for each of these periods is found as follows:

- Period 2: $R_{20}=0.22$
- Period 3: $R_{30}=1.69$
- Period 4: $R_{40}=0.91$
- Period 5: $R_{50}=1.38$

Comparing the basic reproduction numbers for all the five periods, we see that $R_{10}=2.01$ is the highest, which is associated with the initial ascending phase of the epidemic where the infections are typically increasing exponentially. The substantially reduced value of $R_{20}$ for Period 2 is a result of the stay-at-home order in Tennessee that was implemented in April 2020, leading to a very low level of social contacts and disease transmission risk at the beginning of Period 2. As the stay-at-home order expired, businesses were re-opened, and people gradually resumed most of the their normal activities, our model shows that $R_{30}$ bounces back to a high value for Period 3. This is followed by another reduction of the basic reproduction number for Period 4, where $R_{40}$ is slightly below unity, possibly due to the improved public awareness and health management stimulated by the high disease risk in Period 3. Finally, the rebound of $R_{50}$ for Period 5 is a reflection of the second COVID-19 wave that swept through the US during the last few months of 2020. Our simulation result for the evolution of the mitigation level *M* shows a pattern consistent with that of the policy curves in Figure 1 (right).

**Figure 21.**
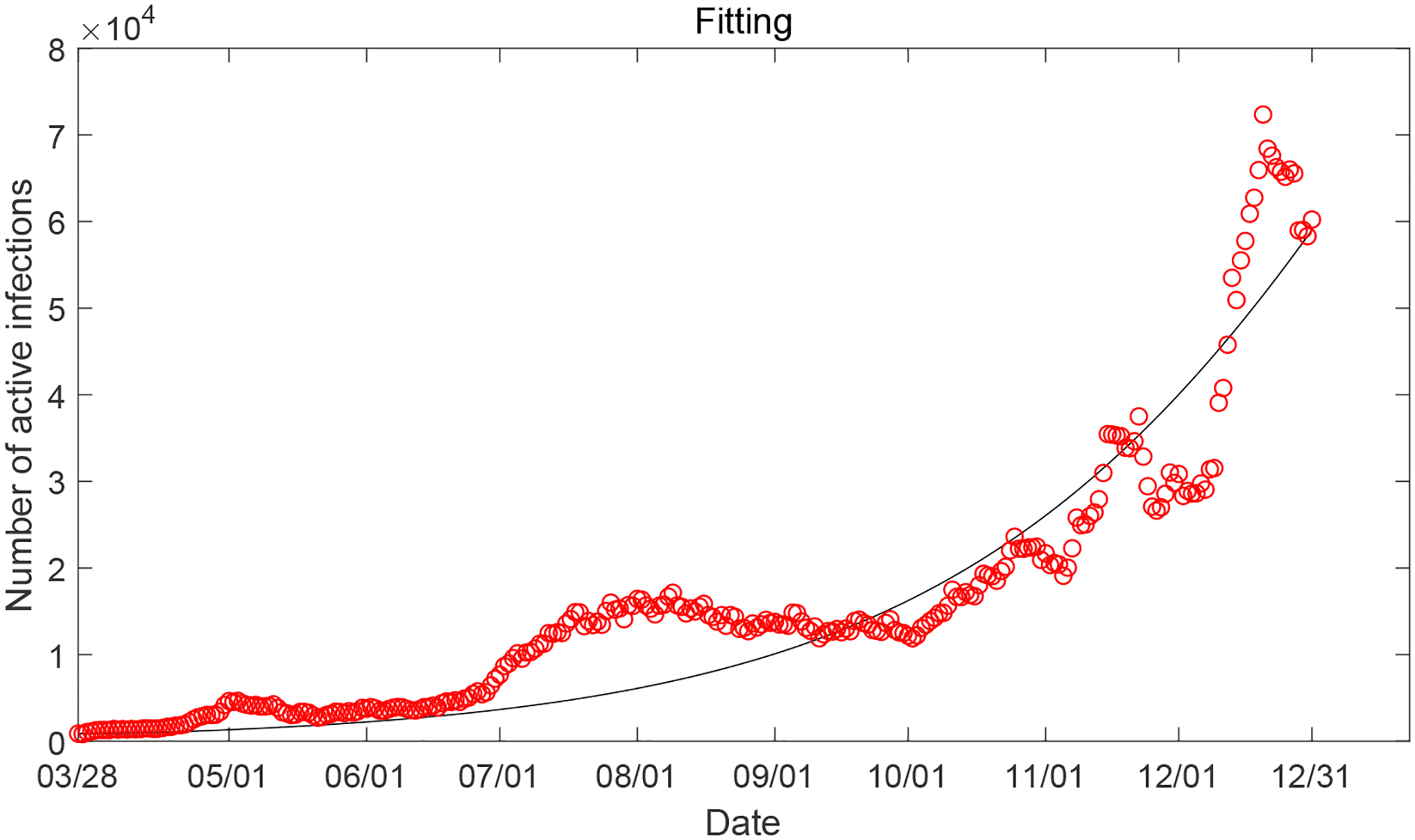
Data fitting for active COVID-19 cases in Tennessee over the entire time interval (from March 28, 2020 to December 31, 2020): circles represent the reported data and solid line represents the fitting result.

In addition, we present the result of data fitting for the entire time interval (from March 28, 2020 to December 31, 2020) in Figure 21, where the values of fitted parameters are given by the last column in Table 2. We see that for several timeframes, some of which even span 1–2 months, our simulation curve is unable to well catch the behavior of the reported data, which provides a justification for our separated fitting and simulation in different periods.

## 4. Discussion

We have presented a new mathematical model to study the interaction between the disease transmission and spread, the epidemic management, and the economic development associated with COVID-19. We have also fitted our model to the reported COVID-19 cases and unemployment rates in the US state of Tennessee to illustrate the application of our modeling framework. We have focused our attention on the model fitting and simulation during the initial period of the COVID-19 epidemic in Tennessee (from March 28 to April 30, 2020), though our simulation study for the remainder of the year 2020 (until December 31) is also summarized. We have considered a few special cases, including the extreme scenarios where there is no mitigation activity (*M* = 0) or there is no economic development (*C* = 0), and compared the different dynamics between those and the realistic scenario where both disease control and economic growth are present. Meanwhile, we have conducted a thorough sensitivity analysis of the model parameters, and utilized detailed simulation results to examine the impact on the model outcome when the values of several sensitive parameters are varied.

Our study represents an application of mathematical modeling and simulation at the interface between epidemiology and economics. The main contributions of this work can be summarized as follows.

### Contribution to economics.

Our findings could provide useful insight into economic development under the impact of COVID-19. Although our model is coarse-grained, simplifying the many factors in disease management and economic development to two variables (*M* and *C*), the simulation results display a rich set of dynamics that highlight the complex interaction involved. Specifically, our numerical findings show several typical patterns: (i) increased disease prevalence stimulates stronger mitigation strategies; (ii) a higher level of disease control leads to reduced economic growth (measured by a higher unemployment rate); (iii) a lower level of economic development could help to slow down the epidemic spread, but the demand for economic recovery would tend to weaken the pandemic management and increase the risk of infection; (iv) within the triad of the epidemic progression, public health management, and economic development, the change of any single component will impact the other two, though such an impact may not be immediately noticeable and may often be seen in a delayed manner. These observations indicate the importance of striking a balance between the disease control and economic growth during the pandemic era, and could provide useful guidelines toward decision making and policy development.

### Contribution to epidemic modeling.

Our work represents a new modeling study in the epidemiology of COVID-19. Under a homogeneous setting, our mathematical analysis (presented in Appendix B) indicates that if $R_{0}>1$, the disease would persist in the long term and the stable endemic equilibrium could potentially have a basin of attraction in the entire domain. Practically, however, public health management and change of human behavior could reduce the value of $R_{0}$ below unity, so that the disease may be eradicated in a population. Even if this is not possible, strategical control measures could minimize the impact of the endemism, which, mathematically, corresponds to a decrease in the magnitude of the endemic equilibrium and/or a reduction of its basin of attraction to a small region. Furthermore, the interaction between economic growth and disease control may strongly impact the epidemic progression and lead to time-dependent system dynamics, so that the assumption of a homogeneous setting is no longer valid. Specifically, the number of reported COVID-19 cases in Tennessee (and possibly in the entire US as well) from March 2020 to December 2020 exhibits a few distinct features at different times, which has motivated us to divide the whole time interval (which is more than 9 months) into five different periods for data fitting and simulation. This allows us to better fit our model to the real data within each time frame. The simulation results at the end of each time period naturally provide initial conditions to the next period. Though we have focused on the first period, this separated fitting approach would enable us to conduct a detailed investigation of the epidemic-economic dynamics in each different period when needed, and the method can be easily extended to the fitting and simulation in other places and/or times.

Additionally, this modeling study builds a foundation for deeper predictive investigations into the relationship between the pandemic progression, disease control, and economic development. In particular, an interesting and practically meaningful task is to explore the ‘best’ balance between the pandemic management and economic growth. This can be formulated as an optimal control problem [54–56], and several epidemic-economic optimal policy studies have already been performed with various focuses [57–60]. For our modeling investigation, mathematical theory and numerical simulation can be applied to find a possible optimal control solution that could minimize the costs associated with the infection (morbidity and mortality), the control measures (prevention, tests, diagnosis, vaccination, treatment, etc.), and the reduction in economic development (increased unemployment rates, in particular). Such a result would provide helpful guidelines to the government, the public health administration, and other policy makers.

## Figures and Tables

**Table 1.** Definitions of model parameters.

Parameter	Description	Value	Unit	Source
*μ*	Natural death rate	2.74 × 10^−5^	per day	[46]
*λ*	Influx rate of human population	187.1146	person/day	[46]
*ω*	Disease-induced death rate	0.01	per day	[1]
*γ*	Recovery rate	1/14	per day	[1]
*α*	Reciprocal of the incubation period	1/7	per day	[51]
*β*_*E*_	Transmission rate from *E* to *S*	-	/person/day	Estimated
*β*_*I*_	Transmission rate from *I* to *S*	-	/person/day	Estimated
*δ*	Influx rate of disease mitigation	-	per day	Estimated
*m*	Mitigation strength stimulated by *I*	-	/person/day	Estimated
*p*	Natural reduction rate of mitigation	-	per day	Estimated
*f*_0_	Decline rate of mitigation due to economic activities	-	per day	Estimated
*c*_*S*_	Labor contribution rate from *S*	-	/person/day	Estimated
*c*_*E*_	Labor contribution rate from *E*	-	/person/day	Estimated
*c*_*R*_	Labor contribution rate from *R*	-	/person/day	Estimated
*d*	Natural reduction rate of economic development	-	per day	Estimated
*g*_0_	Decline rate of economic growth due to mitigation	-	per day	Estimated
*b*	Implementation rate of disease mitigation	-	-	Estimated
${\bar{S}}_{j0}$	Initial value of the susceptible in Period *j*	-	person	[46]
${\bar{E}}_{j0}$	Initial value of the exposed in Period *j*	-	person	Estimated
${\bar{I}}_{j0}$	Initial value of the infected in Period *j*	-	person	[46]
${\bar{R}}_{j0}$	Initial value of the recovered in Period *j*	-	person	[46]
${\bar{M}}_{j0}$	Initial value of the mitigation level in Period *j*	-	-	Estimated
${\bar{C}}_{j0}$	Initial value of the economic level in Period *j*	-	-	[47]

**Table 2.** Parameter values fitted from Period *j* (1 ≤ *j* ≤ 5) and the entire time interval.

Parameter	*j* = 1	*j* = 2	*j* = 3	*j* = 4	*j* = 5	entire interval
*β*_*E*_	8.99 × 10^−9^	7.0 × 10^−10^	7.39 × 10^−9^	6.32 × 10^−9^	6.30 × 10^−9^	6.05 × 10^−9^
*β*_*I*_	3.99 × 10^−8^	3.0 × 10^−9^	1.85 × 10^−8^	1.13 × 10^−8^	1.35 × 10^−8^	1.49 × 10^−8^
*δ*	0.59	0.3	0.23	0.16	0.23	0.17
*m*	9.0 × 10^−10^	5.0 × 10^−10^	2.50 × 10^−10^	2.88 × 10^−7^	2.34 × 10^−10^	1.64 × 10^−7^
*p*	0.68	0.5	0.61	0.31	0.63	0.81
*f*_0_	1.23 × 10^−9^	3.0 × 10^−9^	5.35 × 10^−9^	3.34 × 10^−6^	6.28 × 10^−9^	4.21 × 10^−4^
*c*_*S*_	2.99 × 10^−8^	3.0 × 10^−8^	3.16 × 10^−8^	3.36 × 10^−8^	3.64 × 10^−8^	2.05 × 10^−8^
*c*_*E*_	1.50 × 10^−8^	2.0 × 10^−9^	4.62 × 10^−9^	8.34 × 10^−9^	5.06 × 10^−9^	1.73 × 10^−8^
*c*_*R*_	2.91 × 10^−8^	3.0 × 10^−7^	2.37 × 10^−8^	2.15 × 10^−8^	2.95 × 10^−8^	3.99 × 10^−8^
*d*	0.228648	0.23172	0.233404	0.221	0.2484	0.15292
*g*_0_	1.44 × 10^−5^	2.59 × 10^−6^	2.80 × 10^−4^	3.28 × 10^−2^	2.51 × 10^−4^	1.42 × 10^−5^
*b*	0.99	0.5	0.34	0.73	0.10	0.87
${\bar{S}}_{j0}$	6, 829, 000	6,818,747	6,817,479	6, 761, 941	6, 688, 381	6, 829, 000
${\bar{E}}_{j0}$	442	3233	542	12, 689	5958	442
${\bar{I}}_{j0}$	883	4640	2802	13, 882	12, 178	883
${\bar{R}}_{j0}$	551	7733	16, 359	67, 257	180, 990	551
${\bar{M}}_{j0}$	0.8	0.88256	0.6	0.375779	0.516058	0.8
${\bar{C}}_{j0}$	0.9885	0.896	0.8985	0.9215	0.9542	0.9885

**Table 3.** Parameter values fitted from Period 1 for three special cases.

Parameter	*M* = 0	*C* = 0	*M* = *C* = 0
*β*_*E*_	6.0 × 10^−9^	8.86 × 10^−9^	1.0 × 10^−9^
*β*_*I*_	2.20 × 10^−8^	2.74 × 10^−8^	2.67 × 10^−8^
*δ*	-	0.40	-
*m*	-	9.19 × 10^−9^	-
*p*	-	0.90	-
*c*_*S*_	9.0 × 10^−8^	-	-
*c*_*E*_	1.0 × 10^−8^	-	-
*c*_*R*_	1.07 × 10^−8^	-	-
*d*	0.90	-	-
*b*	-	0.80	-

**Table 4.** Ranked parameter sensitivity (from the highest to the lowest) for $R_{10}$.

Rank	Parameter	Sensitivity	Rank	Parameter	Sensitivity
1	*λ*	1.000000001	9	*ω*	−0.1088
2	*β*_*I*_	0.8863	10	*d*	4.02 × 10^−5^
3	*γ*	−0.7772	11	*c*_*S*_	8.5884 × 10^−10^
4	*δ*	−0.46875	12	*f*_0_	8.588 × 10^−10^
5	*p*	0.46875	13	*g*_0_	5.312 × 10^−14^
6	*b*	−0.468749	14	*m*	0
7	*β*_*E*_	0.1137	15	*c*_*E*_	0
8	*α*	−0.1135	16	*c*_*R*_	0

## Appendix A: Domain and reproduction number

We first determine a feasible domain for our mathematical model (2.1). If we add up the first four equations in system (2.1), we easily obtain

(A.1)
$$S+E+I+R\leq \frac{\lambda }{\mu }.$$

Meanwhile, adding the first two equations yields

(A.2)
$$S+E\geq \frac{\lambda }{\alpha +\mu }.$$

Next, we derive conditions to ensure 0 ≤ *M* ≤ 1 and 0 ≤ *C* ≤ 1. For *M* ≥ 0, we need $\frac{dM}{dt}\geq 0$ when *M* = 0. This yields *δ* + *mI* − *f*_0_*C* ≥ 0, which holds for all *I* ≥ 0 and *C* ≤ 1 if

(A.3)
$$\delta -f_{0}\geq 0.$$

To ensure *M* ≤ 1, we need $\frac{dM}{dt}\leq 0$ when *M* = 1. This leads to *δ* + *mI* − *p* − *f*_0_*C* ≤ 0, which holds for all $I\leq \frac{\lambda }{\mu }$ and *C* ≥ 0 if

(A.4)
$$\delta +m\frac{\lambda }{\mu }-p\leq 0.$$

With similar arguments, we can make certain *C* ≥ 0 if

(A.5)
$$c_{m}\frac{\lambda }{\alpha +\mu }-g_{0}\geq 0,\text{ where }c_{m}=\text{min}\left(c_{S},c_{E},c_{R}\right),$$

by using condition (A.2), and ensure *C* ≤ 1 if

(A.6)
$$c_{M}\frac{\lambda }{\mu }-d\leq 0,\text{ where }c_{M}=\text{max}\left(c_{S},c_{E},c_{R}\right),$$

by using condition (A.1).

Thus, under conditions (A.3)–(A.6), the biologically feasible domain

(A.7)
$$\Gamma =\left\{(S,E,I,R,M,C):S,E,I,R\geq 0,S+E+I+R\leq \frac{\lambda }{\mu },0\leq M\leq 1,0\leq C\leq 1\right\}$$

is invariant with respect to the vector field of system (2.1).

Obviously, there is a unique disease-free equilibrium (DFE) of system (2.1) which is given by

(A.8)
$$x_{0}=\left(S_{0},0,0,0,M_{0},C_{0}\right)=\left(\frac{\lambda }{\mu },0,0,0,\frac{d\delta -f_{0}c_{S}S_{0}}{dp-g_{0}f_{0}},\frac{pc_{S}S_{0}-g_{0}\delta }{dp-g_{0}f_{0}}\right).$$

Using conditions (A.3) and (A.6), we have $d\delta -f_{0}c_{S}S_{0}\geq f_{0}\left(d-c_{S}\frac{\lambda }{\mu }\right)\geq f_{0}\left(c_{M}\frac{\lambda }{\mu }-c_{S}\frac{\lambda }{\mu }\right)\geq 0$. Meanwhile, conditions (A.3) and (A.4) yield *p* > *δ* ≥ *f*_0_, and conditions (A.5) and (A.6) yield $d\geq c_{M}\frac{\lambda }{\mu }>c_{m}\frac{\lambda }{\alpha +\mu }\geq g_{0}$. Hence, *dp*−*g*_0_ *f*_0_ > 0, and $pc_{S}S_{0}-g_{0}\delta >\delta \left(c_{S}S_{0}-g_{0}\right)>\delta \left(c_{m}\frac{\lambda }{\alpha +\mu }-g_{0}\right)\geq 0$. These ensure that *M*_0_ ≥ 0 and *C*_0_ > 0 in equation (A.8). Moreover, conditions (A.4) and (A.5) yield *dδ* − *f*_0_*c*_*S*_ *S*_0_ < *dp* − *g*_0_ *f*_0_, or *M*_0_ < 1, and conditions (A.3) and (A.6) yield *pc*_*S*_ *S*_0_ − *g*_0_*δ* ≤ *dp* − *g*_0_ *f*_0_, or *C*_0_ ≤ 1. Therefore, the DFE *x*_0_ is within the domain Γ.

Using the DFE, we can evaluate the basic reproduction number of model (2.1) based on the standard next-generation matrix approach [61]. The new infection matrix *F* and the transition matrix *V* are

(A.9)
$$F=\left(\begin{array}{cc} \frac{\beta_{E}S_{0}}{1+bM_{0}} & \frac{\beta_{I}S_{0}}{1+bM_{0}}\\ 0 & 0 \end{array}\right),\text{ and }V=\left(\begin{array}{cc} \alpha +\mu & 0\\ -\alpha & w+\gamma +\mu \end{array}\right).$$

Thus, the next-generation matrix is given as

(A.10)
$$FV^{-1}=\left(\begin{array}{cc} a_{11} & a_{12}\\ 0 & 0 \end{array}\right),$$

where $a_{11}=\frac{\beta_{E}S_{0}}{\left(1+bM_{0}\right)(\alpha +\mu )}+\frac{\alpha \beta_{I}S_{0}}{\left(1+bM_{0}\right)(\alpha +\mu )(w+\gamma +\mu )}$ and $a_{12}=\frac{\beta_{I}S_{0}}{\left(1+bM_{0}\right)(w+\gamma +\mu )}$.

The basic reproduction number is then computed by the spectral radius of the next-generation matrix: $R_{0}=\rho \left(FV^{-1}\right)$; i.e.,

(A.11)
$$R_{0}=\frac{\beta_{E}S_{0}}{\left(1+bM_{0}\right)(\alpha +\mu )}+\frac{\alpha \beta_{I}S_{0}}{\left(1+bM_{0}\right)(\alpha +\mu )(w+\gamma +\mu )}.$$

## Appendix B: Equilibrium analysis

Here we analyze the equilibrium points of system (2.1) and their stability properties. We have determined the unique disease-free equilibrium in Appendix A. Now we establish the following result regarding the existence and uniqueness of the endemic equilibrium.

**Theorem B.1.** *Assume mλ* − *f*_0_*γ* ≥ 0. *When $R_{0}>1$*, *there exists a unique endemic equilibrium x*_*_ = (*S*_*_, *E*_*_, *I*_*_, *R*_*_, *M*_*_, *C*_*_) *defined by* Eqs (B.7)–(B.12).

*Proof*. At an endemic equilibrium of system (2.1), we have

(B.1)
$$\lambda -\frac{\beta_{E}}{1+bM_{*}}S_{*}E_{*}-\frac{\beta_{I}}{1+bM_{*}}S_{*}I_{*}-\mu S_{*}=0,$$

(B.2)
$$\frac{\beta_{E}}{1+bM_{*}}S_{*}E_{*}+\frac{\beta_{I}}{1+bM_{*}}S_{*}I_{*}-(\alpha +\mu )E_{*}=0,$$

(B.3)
$$\alpha E_{*}-(w+\gamma +\mu )I_{*}=0,$$

(B.4)
$$\gamma I_{*}-\mu R_{*}=0,$$

(B.5)
$$\delta +mI_{*}-pM_{*}-f_{0}C_{*}=0,$$

(B.6)
$$c_{S}S_{*}+c_{E}E_{*}+c_{R}R_{*}-dC_{*}-g_{0}M_{*}=0.$$

Adding (B.1) to (B.2), we obtain

(B.7)
$$S_{*}=S_{0}-\frac{\alpha +\mu }{\mu }E_{*}.$$

By Eqs (B.3) and (B.4), we have

(B.8)
$$I_{*}=\frac{\alpha }{w+\gamma +\mu }E_{*}$$

and

(B.9)
$$R_{*}=\frac{\gamma }{\mu }\frac{\alpha }{w+\gamma +\mu }E_{*}.$$

Using Eqs (B.5) and (B.6), we have

(B.10)
$$M_{*}=\frac{mdI+d\delta -f_{0}\left(c_{S}S_{*}+c_{E}E_{*}+c_{R}R_{*}\right)}{pd-f_{0}g_{0}}$$

and

(B.11)
$$C_{*}=\frac{p\left(c_{S}S_{*}+c_{E}E_{*}+c_{R}R_{*}\right)-g_{0}mI-g_{0}\delta }{pd-f_{0}g_{0}}.$$

Substituting Eqs (B.7)–(B.11) to (B.2), we obtain a unique non-trivial solution for *E*_*_:

(B.12)
$$E_{*}=\frac{\beta_{E}S_{0}+\beta_{I}S_{0}\cdot \frac{\alpha }{w+\gamma +\mu }+\frac{b(\alpha +\mu )\left(f_{0}c_{S}S_{0}-\delta d\right)}{dp-f_{0}g_{0}}-(\alpha +\mu )}{\beta_{E}\cdot \frac{\alpha +\mu }{\mu }+\beta_{I}\cdot \frac{\alpha }{w+\gamma +\mu }\cdot \frac{\alpha +\mu }{\mu }+\frac{(\alpha +\mu )bdm\cdot \frac{\alpha }{w+\gamma +\mu }+(\alpha +\mu )bf_{0}\left(c_{S}\cdot \frac{\alpha +\mu }{\mu }-c_{E}-c_{R}\cdot \frac{\gamma }{\mu }\cdot \frac{\alpha }{w+\gamma +\mu }\right)}{dp-f_{0}g_{0}}}\;=\frac{\left(1+bM_{0}\right)\left(R_{0}-1\right)}{\frac{\left(\alpha +\mu \right)}{\lambda }\left(1+bM_{0}\right)\left(R_{0}-1\right)+\frac{b}{dp-f_{0}g_{0}}\left(md\cdot \frac{\alpha }{w+\gamma +\mu }-f_{0}c_{E}-f_{0}c_{R}\cdot \frac{\gamma }{\mu }\cdot \frac{\alpha }{w+\gamma +\mu }\right)+\frac{\alpha +\mu }{\lambda }\left(1+\frac{bd\delta }{dp-f_{0}g_{0}}\right)}\;=\frac{\left(1+bM_{0}\right)\left(R_{0}-1\right)}{\frac{\left(\alpha +\mu \right)}{\lambda }\left(1+bM_{0}\right)\left(R_{0}-1\right)+\frac{\alpha +\mu }{\lambda }+A},$$

where we may re-write *A* as

(B.13)
$$A=\frac{b}{dp-f_{0}g_{0}}\left(d\delta \frac{\alpha +\mu }{\lambda }-f_{0}c_{E}\right)+\frac{b}{dp-f_{0}g_{0}}\frac{\alpha }{w+\gamma +\mu }\left(md-f_{0}c_{R}\frac{\gamma }{\mu }\right).$$

We know *dp* − *f*_0_*g*_0_ > 0 from Eq (A.8) and the analysis afterwards. Using conditions (A.3) and (A.6), we have $d\delta \frac{\alpha +\mu }{\lambda }-f_{0}c_{E}\geq c_{M}\frac{\lambda }{\mu }\cdot \delta \frac{\alpha +\mu }{\lambda }-f_{0}c_{E}>c_{M}\delta -f_{0}c_{E}\geq 0$. Meanwhile, using the assumption *mλ* − *f*_0_*γ* ≥ 0, we obtain $md-f_{0}c_{R}\frac{\gamma }{\mu }\geq mc_{M}\frac{\lambda }{\mu }-f_{0}c_{R}\frac{\gamma }{\mu }\geq \frac{c_{R}}{\mu }\left(m\lambda -f_{0}\gamma \right)\geq 0$. Hence, *A* > 0, which yields *E*_*_ > 0 when $R_{0}>1$. Consequently, there is a unique endemic equilibrium *x*_*_ when $R_{0}>1$.

Next, we consider the stability properties of the DFE and endemic equilibrium. Based on the standard theory of the basic reproduction number [61], the DFE is locally asymptotically stable when $R_{0}<1$ and becomes unstable when $R_{0}>1$. We proceed to study the stability of the endemic equilibrium near the bifurcation point $R_{0}=1$ using local bifurcation analysis [62]. We first introduce the following lemma.

**Lemma B.1.** *(* [61], *Theorem 4) Consider the disease transmission model defined by ẋ* = *f*(*x*, *μ*)*, where μ is a bifurcation parameter such that* $R_{0}<1$ *for μ* < 0 *and* $R_{0}>1$ *for μ* > 0 *and such that x*_0_ *is a DFE for all values of μ. Assume that the zero eigenvalue of D*_*x*_ *f*(*x*_0_, 0) *is simple, where D*_*x*_ *f*(*x*_0_, 0) *is the partial derivative of f with respect to x evaluated at the point x* = *x*_0_*, μ* = 0*, and let v and ω be the corresponding left and right nullvectors chosen such that vω* = 1*. Let a and b be defined as follows:*

(B.14)
$$a=\frac{v}{2}D_{xx}f\left(x_{0},0\right)\omega^{2}=\frac{1}{2}\overset{n}{\underset{i,j,k=1}{\sum }}v_{i}\omega_{j}\omega_{k}\frac{\partial^{2}f_{i}}{\partial x_{j}\partial x_{k}}\left(x_{0},0\right),$$

(B.15)
$$b=vD_{x\mu }f\left(x_{0},0\right)\omega =\overset{n}{\underset{i,j=1}{\sum }}v_{i}\omega_{j}\frac{\partial^{2}f_{i}}{\partial x_{j}\partial \mu }\left(x_{0},0\right),$$

*and assume that b* ≠ 0*. Also assume that the DFE is stable when μ* < 0*. Then b* > 0*, and there exists ε* > 0 *such that*

- *if a* < 0, *there are locally asymptotically stable endemic equilibria near x*_0_ *for* 0 < *μ* < *ε*;
- *if a* > 0, *there are unstable endemic equilibria near x*_0_ *for* −*ε* < *μ* < 0.

We utilize Lemma B.1 to prove the result below.

**Theorem B.2.** *When* $R_{0}>1$ *and* $R_{0}-1$ *is sufficiently small, the endemic equilibrium x*_*_ *of system* (2.1) *is locally asymptotically stable*.

*Proof*. Let us re-write system (2.1) as $\dot{x}=f\left(x,R_{0}\right)$, where *x* = [*x*_1_, *x*_2_, *x*_3_, *x*_4_, *x*_5_, *x*_6_]^*T*^ = [*S*, *E*, *I*, *R*, *M*, *C*]^*T*^ and *f* = [*f*_1_, *f*_2_, *f*_3_, *f*_4_, *f*_5_, *f*_6_]^*T*^ = [*Ṡ*, *Ė*, *İ*, *Ṙ*, *Ṁ*, *Ċ*]^*T*^.

The Jacobian matrix of system (2.1) at (*x*_0_, 1) is given by

$$D_{x}f\left(x_{0},1\right)=\left(\begin{array}{cccccc} -\mu & \frac{-\beta_{E}S_{0}}{1+bM_{0}} & \frac{-\beta_{I}S_{0}}{1+bM_{0}} & 0 & 0 & 0\\ 0 & \frac{\beta_{E}S_{0}}{1+bM_{0}}-(\alpha +\mu ) & \frac{\beta_{I}S_{0}}{1+bM_{0}} & 0 & 0 & 0\\ 0 & \alpha & -(\omega +\gamma +\mu ) & 0 & 0 & 0\\ 0 & 0 & \gamma & -\mu & 0 & 0\\ 0 & 0 & m & 0 & -p & -f_{0}\\ c_{S} & c_{E} & 0 & c_{R} & -g_{0} & -d \end{array}\right).$$

Let $R_{0}=1$. We manipulate the characteristic polynomial as follows:

$$\text{det}\left[\lambda I-D_{x}f\left(x_{0},1\right)\right]=-\left[\lambda^{2}+(p+d)\lambda +pd-f_{0}g_{0}\right]{\left(\lambda +\mu \right)}^{2}\cdot \left\{\left[\lambda -\frac{\beta_{E}S_{0}}{1+bM_{0}}+(\alpha +\mu )\right](\lambda +w+\gamma +\mu )-\frac{\alpha \beta_{I}S_{0}}{1+bM_{0}}\right\}\;=-\left[\lambda^{2}+(p+d)\lambda +pd-f_{0}g_{0}\right]{\left(\lambda +\mu \right)}^{2}\cdot \left\{\lambda^{2}-\lambda \left[\frac{\beta_{E}S_{0}}{1+bM_{0}}-(\alpha +\mu )-(w+\gamma +\mu )\right]-(w+\gamma +\mu )(\alpha +\mu )\left(R_{0}-1\right)\right\}\;=-\left[\lambda^{2}+(p+d)\lambda +pd-f_{0}g_{0}\right]{\left(\lambda +\mu \right)}^{2}\cdot \lambda \left\{\lambda -\left[\frac{\beta_{E}S_{0}}{1+bM_{0}}-(\alpha +\mu )-(w+\gamma +\mu )\right]\right\}.$$

Note that $R_{0}$ is defined in Eq (A.11). We define

$$R_{01}=\frac{\beta_{E}S_{0}}{\left(1+bM_{0}\right)(\alpha +\mu )},\;R_{02}=\frac{\alpha \beta_{I}S_{0}}{\left(1+bM_{0}\right)(\alpha +\mu )(w+\gamma +\mu )}.$$

Since $R_{01}<R_{0}=1$, we have

$$\frac{\beta_{E}S_{0}}{1+bM_{0}}-(\alpha +\mu )-(w+\gamma +\mu )=(\alpha +\mu )\left[R_{01}-1-\frac{w+\gamma +\mu }{\alpha +\mu }\right]<0.$$

Since *pd* − *f*_0_*g*_0_ > 0, we see that the zero eigenvalue of *D*_*x*_ *f*(*x*_0_, 1) is simple and all other eigenvalues of *D*_*x*_ *f*(*x*_0_, 1) have negative real parts.

All the second derivatives of *f*_*i*_ in (B.14) are zero at the DFE except the following:

$$\frac{\partial^{2}f_{1}}{\partial S\partial E}\left(x_{0},1\right)=\frac{-\beta_{E}}{1+bM_{0}},\;\frac{\partial^{2}f_{1}}{\partial S\partial I}\left(x_{0},1\right)=\frac{-\beta_{L}}{1+bM_{0}},\;\frac{\partial^{2}f_{1}}{\partial E\partial S}\left(x_{0},1\right)=\frac{-\beta_{E}}{1+bM_{0}},\;\frac{\partial^{2}f_{1}}{\partial L\partial S}\left(x_{0},1\right)=\frac{-\beta_{I}}{1+bM_{0}},$$

$$\frac{\partial^{2}f_{1}}{\partial E\partial M}\left(x_{0},1\right)=\frac{\beta_{E}S_{0}b}{{\left(1+bM_{0}\right)}^{2}},\;\frac{\partial^{2}f_{1}}{\partial IM}\left(x_{0},1\right)=\frac{\beta_{I}S_{0}b}{{\left(1+bM_{0}\right)}^{2}},\;\frac{\partial^{2}f_{1}}{\partial M\partial E}\left(x_{0},1\right)=\frac{\beta_{E}S_{0}b}{{\left(1+bM_{0}\right)}^{2}},\;\frac{\partial^{2}f_{1}}{\partial M\partial I}\left(x_{0},1\right)=\frac{\beta_{I}S_{0}b}{{\left(1+bM_{0}\right)}^{2}},$$

$$\frac{\partial^{2}f_{2}}{\partial S\partial E}\left(x_{0},1\right)=\frac{\beta_{E}}{1+bM_{0}},\;\frac{\partial^{2}f_{2}}{\partial S\partial I}\left(x_{0},1\right)=\frac{\beta_{I}}{1+bM_{0}},\;\frac{\partial^{2}f_{2}}{\partial E\partial S}\left(x_{0},1\right)=\frac{\beta_{E}}{1+bM_{0}},\;\frac{\partial^{2}f_{2}}{\partial I\partial S}\left(x_{0},1\right)=\frac{\beta_{I}}{1+bM_{0}},$$

$$\frac{\partial^{2}f_{2}}{\partial E\partial M}\left(x_{0},1\right)=\frac{-\beta_{E}S_{0}b}{{\left(1+bM_{0}\right)}^{2}},\;\frac{\partial^{2}f_{2}}{\partial I\partial M}\left(x_{0},1\right)=\frac{-\beta_{1}S_{0}b}{{\left(1+bM_{0}\right)}^{2}},\;\frac{\partial^{2}f_{2}}{\partial M\partial E}\left(x_{0},1\right)=\frac{-\beta_{E}S_{0}b}{{\left(1+bM_{0}\right)}^{2}},$$

$$\frac{\partial^{2}f_{2}}{\partial M\partial I}\left(x_{0},1\right)=\frac{-\beta_{I}S_{0}b}{{\left(1+bM_{0}\right)}^{2}}.$$

Corresponding first derivatives of *f*_*i*_ in (B.15) are listed as follows:

$$\frac{\partial f_{1}}{\partial E}\left(x_{0},1\right)=\frac{-\beta_{E}S_{0}}{1+bM_{0}}=-(\alpha +\mu )R_{01}=-(\alpha +\mu )\left(R_{0}-R_{02}\right),$$

$$\frac{\partial f_{1}}{\partial I}\left(x_{0},1\right)=\frac{-\beta_{I}S_{0}}{1+bM_{0}}=-\frac{\left(\alpha +\mu )(w+\gamma +\mu \right)}{\alpha }R_{02}=-\frac{\left(\alpha +\mu )(w+\gamma +\mu \right)}{\alpha }\left(R_{0}-R_{01}\right),$$

$$\frac{\partial f_{2}}{\partial E}\left(x_{0},1\right)=\frac{\beta_{E}S_{0}}{1+bM_{0}}-(\alpha +\mu )=(\alpha +\mu )\left(R_{01}-1\right)=(\alpha +\mu )\left(R_{0}-R_{02}-1\right),$$

$$\frac{\partial f_{2}}{\partial I}\left(x_{0},1\right)=\frac{\beta_{I}S_{0}}{1+bM_{0}}=\frac{\left(\alpha +\mu )(w+\gamma +\mu \right)}{\alpha }R_{02}=\frac{\left(\alpha +\mu )(w+\gamma +\mu \right)}{\alpha }\left(R_{0}-R_{01}\right).$$

The non-zero second derivatives of *f*_*i*_ in (B.15) are

$$\frac{\partial^{2}f_{1}}{\partial E\partial R_{0}}\left(x_{0},1\right)=-(\alpha +\mu ),\;\frac{\partial^{2}f_{1}}{\partial I\partial R_{0}}\left(x_{0},1\right)=-\frac{\left(\alpha +\mu )(w+\gamma +\mu \right)}{\alpha },$$

$$\frac{\partial^{2}f_{2}}{\partial E\partial R_{0}}\left(x_{0},1\right)=\alpha +\mu ,\;\frac{\partial^{2}f_{2}}{\partial I\partial R_{0}}\left(x_{0},1\right)=\frac{\left(\alpha +\mu )(w+\gamma +\mu \right)}{\alpha }.$$

We choose *v* and *ω* such that they are orthogonal to *D*_*x*_ *f*(*x*_0_, 1) (i.e., *v* · *D*_*x*_ *f*(*x*_0_,1 ) = 0, *D*_*x*_ *f*(*x*_0_, 1) · *ω* = 0), and *v* · *ω* = 1. With some algebraic manipulation, we obtain *v* = (0, *v*_2_, *v*_3_, 0, 0, 0), where $v_{2}=\frac{1}{1+\frac{\alpha \beta_{I}S_{0}}{1+bM_{0}}}>0$, $v_{3}=\frac{\frac{\beta_{E}S_{0}}{1+bM_{0}}-(\alpha +\mu )}{-\alpha }v_{2}=\frac{\beta_{I}S_{0}}{\left(1+bM_{0}\right)(w+\gamma +\mu )}v_{2}$ due to $R_{0}=1$; and *ω* = (*ω*_1_, *ω*_2_, *ω*_3_, *ω*_4_, *ω*_5_, *ω*_6_), where $\omega_{1}=-\frac{\alpha +\mu }{\mu }<0$, *ω*_2_ = 1, $\omega_{3}=\frac{\alpha }{w+\gamma +\mu }>0$, $\omega_{4}=\frac{\gamma \alpha }{\mu (w+\gamma +\mu )}$, $\omega_{5}=\frac{f_{0}c_{S}\frac{\alpha +\mu }{u}}{pd-f_{0}g_{0}}+\frac{md\cdot \frac{\alpha }{w+\gamma +\mu }-f_{0}c_{E}-f_{0}c_{R}\cdot \frac{\gamma }{\mu }\cdot \frac{\alpha }{w+\gamma +\mu }}{pd-f_{0}g_{0}}>0$, $\omega_{6}=\frac{-pc_{S}\frac{\alpha +\mu }{\mu }-g_{0}\frac{m\alpha }{w+\gamma +\mu }+pc_{E}+pc_{R}\frac{\gamma \alpha }{\mu (w+\gamma +\mu )}}{pd-f_{0}g_{0}}$.

Hence, Eq (B.14) yields

(B.16)
$$a=v_{2}\left[\omega_{1}\omega_{2}\frac{\beta_{E}}{1+bM_{0}}+\omega_{1}\omega_{3}\frac{\beta_{I}}{1+bM_{0}}+\omega_{2}\omega_{5}\frac{-\beta_{E}S_{0}b}{{\left(1+bM_{0}\right)}^{2}}+\omega_{3}\omega_{5}\frac{-\beta_{I}S_{0}b}{{\left(1+bM_{0}\right)}^{2}}\right]<0.$$

Based on Eq (B.15), we can also verify that $b=v_{2}(\alpha +\mu )\left(\omega_{2}+\omega_{3}\frac{w+\gamma +\mu }{\alpha }\right)>0$. Therefore, we conclude that when $R_{0}-1$ changes from negative to positive, a positive and locally asymptotically stable equilibrium *x*_*_ occurs.

Due to the high dimension and strong nonlinearity of system (2.1), we have not fully resolved the global stability of the DFE and the endemic equilibrium. Nevertheless, for a special case where the variable *M* is fixed at any value between 0 and 1, we are able to prove the global asymptotic stability of the simplified system. For illustration, let us substitute *M* = *M*_*_ into system (2.1). Consequently, the basic reproduction number for the reduced system is given by

(B.17)
$$R_{0}=\rho \left(F_{*}V_{*}^{-1}\right)=\frac{\beta_{E}S_{0}}{\left(1+bM_{*}\right)(\alpha +\mu )}+\frac{\alpha \beta_{I}S_{0}}{\left(1+bM_{*}\right)(\alpha +\mu )(w+\gamma +\mu )},$$

where

(B.18)
$$F_{*}=\left(\begin{array}{cc} \frac{\beta_{E}S_{0}}{1+bM_{*}} & \frac{\beta_{I}S_{0}}{1+bM_{*}}\\ 0 & 0 \end{array}\right),\text{ and }V_{*}=\left(\begin{array}{cc} \alpha +\mu & 0\\ -\alpha & w+\gamma +\mu \end{array}\right).$$

**Theorem B.3.** *Let M* = *M*_*_*. When $R_{0}<1$*, *the DFE x*_0_ *of the reduced system from* (2.1) *is globally asymptotically stable*.

*Proof*. When *M* = *M*_*_, the second and third equations of (2.1) imply that

(B.19)
$$\frac{dE}{dt}\leq \frac{\beta_{E}S_{0}E}{1+bM_{*}}+\frac{\beta_{I}S_{0}I}{1+bM_{*}}-(\alpha +\mu )E,\;\frac{dI}{dt}\leq \alpha E-(w+\gamma +\mu )I.$$

Let *Y* = (*E*, *I*). Then system (B.19) yields

(B.20)
$$\frac{dY}{dt}\leq \left(F_{*}-V_{*}\right)Y,$$

where the matrices *F*_*_ and *V*_*_ are given in Eq (B.18).

By the Perron-Frobenius theorem, there exists a positive left eigenvector *u* of the positive matrix $V_{*}^{-1}F_{*}$ with respect to the eigenvalue $R_{0}=\rho \left(F_{*}V_{*}^{-1}\right)=\rho \left(V_{*}^{-1}F_{*}\right)$. Define the Lyapunov function

$$L=u^{T}V_{*}^{-1}Y.$$

Note that *L* ≥ 0, and *L* = 0 if and only if *Y* = 0. Differentiating *L* along the solution of the reduced system from (2.1), we obtain

$$L^{\prime }=u^{T}V_{*}^{-1}\frac{dY}{dt}\leq u^{T}V_{*}^{-1}\left(F_{*}-V_{*}\right)Y=\left(R_{0}-1\right)u^{T}Y.$$

If $R_{0}<1$, then *L*′ ≤ 0. Furthermore, *L*′ = 0 leads to *u*^*T*^*Y* = 0, which yields *E* = *I* = 0. Hence, the largest invariant set where *L*′ = 0 is the DFE *x*_0_. By LaSalle’s Invariance Principle, the DFE is globally asymptotically stable.

**Theorem B.4.** *Let M* = *M*_*_*. When $R_{0}>1$*, *the unique endemic equilibrium x*_*_ *of the reduced system from* (2.1) *is globally asymptotically stable*.

*Proof*. When *M* = *M*_*_, we consider the first three equations of (2.1):

(B.21)
$$\frac{dS}{dt}=\lambda -\frac{\beta_{E}SE}{1+bM_{*}}-\frac{\beta_{I}SI}{1+bM_{*}}-\mu S,\;\frac{dE}{dt}=\frac{\beta_{E}SE}{1+bM_{*}}+\frac{\beta_{I}SI}{1+bM_{*}}-(\alpha +\mu )E,\;\frac{dI}{dt}=\alpha E-(w+\gamma +\mu )I.$$

We introduce the following Lyapunov function:

(B.22)
$$L_{1}(S,E,I)=\int_{S_{*}}^{S}\frac{u-S_{*}}{u}du+\int_{E_{*}}^{E}\frac{u-E_{*}}{u}du+\frac{\beta_{I}S_{*}I_{*}}{\left(1+bM_{*}\right)\alpha E_{*}}\int_{I_{*}}^{I}\frac{u-I_{*}}{u}du.$$

It is easy to verify that *L*_1_ ≥ 0 and *L*_1_ = 0 if and only if (*S*, *E*, *I*) = (*S*_*_, *E*_*_, *I*_*_). Meanwhile, the following equations hold:

(B.23)
$$\lambda -\frac{\beta_{E}S_{*}E_{*}}{1+bM_{*}}-\frac{\beta_{I}S_{*}I_{*}}{1+bM_{*}}-\mu S_{*}=0,$$

(B.24)
$$\frac{\beta_{E}S_{*}E_{*}}{1+bM_{*}}+\frac{\beta_{I}S_{*}I_{*}}{1+bM_{*}}-(\alpha +\mu )E_{*}=0,$$

(B.25)
$$\alpha E_{*}-(w+\gamma +\mu )I_{*}=0.$$

Using Eqs (B.23)–(B.25), we calculate the derivative of *L*_1_ along the solution of the subsystem (B.21) in what follows:

$${L_{1}^{\prime }|}_{\left(\text{B}.21\right)}=\left(1-\frac{S_{*}}{S}\right)\left[\lambda -\frac{\beta_{E}}{1+bM_{*}}SE-\frac{\beta_{I}}{1+bM_{*}}SI-\mu S-\left(\lambda -\frac{\beta_{E}S_{*}E_{*}}{1+bM_{*}}-\frac{\beta_{I}S_{*}I_{*}}{1+bM_{*}}-\mu S_{*}\right)\right]\;+\left(1-\frac{E_{*}}{E}\right)\left\{\left[\frac{\beta_{E}}{1+bM_{*}}SE+\frac{\beta_{I}}{1+bM_{*}}SI-(\alpha +\mu )E\right]-\left[\frac{\beta_{E}S_{*}E_{*}}{1+bM_{*}}+\frac{\beta_{I}S_{*}I_{*}}{1+bM_{*}}-(\alpha +\mu )E_{*}\right]\right\}\;+\frac{\beta_{I}S_{*}I_{*}}{\left(1+bM_{*}\right)\alpha E_{*}}\left(1-\frac{I_{*}}{I}\right)\left\{[\alpha E-(w+\gamma +\mu )I]-\left[\alpha E_{*}-(w+\gamma +\mu )I_{*}\right]\right\}\;=\frac{\beta_{E}}{1+bM_{*}}S_{*}E_{*}(1-\frac{SE}{S_{*}E_{*}}-\frac{S_{*}}{S}+\frac{\overset{}{\overset{︷}{E}}}{E_{*}})+\frac{\beta_{I}}{1+bM_{*}}S_{*}I_{*}(\underset{}{\underset{︸}{1-\frac{SI}{S_{*}I_{*}}}}-\frac{S_{*}}{S}+\frac{\hat{I}}{I_{*}})\;+\mu S_{*}\left(2-\frac{S}{S_{*}}-\frac{S_{*}}{S}\right)+\frac{\beta_{E}}{1+bM_{*}}S_{*}E_{*}(\underline{-1+\frac{SE}{S_{*}E_{*}}}-\frac{S}{S_{*}}+\frac{\overset{}{\overset{︷}{E_{*}}}}{E})\;+\frac{\beta_{I}}{1+bM_{*}}S_{*}I_{*}(\underset{}{\underset{︸}{-1+\frac{SI}{S_{*}I_{*}}}}-\frac{E_{*}SI}{ES_{*}^{T}I_{*}}+\frac{E_{*}}{\underline{\underline{E}}})+\frac{\beta_{E}}{1+bM_{*}}S_{*}E_{*}^{T}(2-\overset{}{\overset{︷}{\frac{E_{*}}{E}-\frac{E}{E_{*}}}})\;+\frac{\beta_{I}}{1+bM_{*}}S_{*}I_{*}\left(2-\underline{\underline{\frac{E_{*}}{E}-\frac{E}{E_{*}}}}\right)\;+\frac{\beta_{I}}{1+bM_{*}}S_{*}I_{*}\left(\frac{E}{\underline{\underline{E_{*}}}}-1-\frac{EI_{*}}{E_{*}I}+\frac{\hat{I_{*}}}{I}\right)+\frac{\beta_{I}}{1+bM_{*}}S_{*}I_{*}\left(2-\frac{\hat{I}}{I_{*}}-\frac{I_{*}}{I}\right)\;=\frac{\beta_{E}}{1+bM_{*}}S_{*}E_{*}\left(2-\frac{S}{S_{*}}-\frac{S_{*}}{S}\right)+\mu S_{*}\left(2-\frac{S}{S_{*}}-\frac{S_{*}}{S}\right)+\frac{\beta_{I}}{1+bM_{*}}S_{*}I_{*}\left(3-\frac{S_{*}}{S}-\frac{EI_{*}}{E_{*}I}-\frac{E_{*}SI}{ES_{*}I_{*}}\right)\;\leq 0,$$

where those terms marked with the same type of symbols are canceled out. It is clear that ${L_{1}^{\prime }|}_{\left(\text{B}.21\right)}=0$ if and only if (*S*, *E*, *I*) = (*S*_*_, *E*_*_, *I*_*_). Hence, according to LaSalle’s Invariance Principle, the endemic equilibrium (*S*_*_, *E*_*_, *I*_*_) of subsystem (B.21) is globally asymptotically stable.

Through substitution of (*S*_*_, *E*_*_, *I*_*_) into the equations for *R* and *C*:

(B.26)
$$\frac{dR}{dt}=\gamma I_{*}-\mu R,\;\frac{dC}{dt}=c_{S}S_{*}+c_{E}E_{*}+c_{R}R-dC-g_{0}M_{*},$$

it is straightforward to observe that (*R*_*_, *C*_*_) of subsystem (B.26) is globally asymptotically stable. The proof is then completed by combining the results for subsystems (B.21) and (B.26).
